# Tenuous Transcriptional Threshold of Human Sex Determination. I. SRY and Swyer Syndrome at the Edge of Ambiguity

**DOI:** 10.3389/fendo.2022.945030

**Published:** 2022-07-26

**Authors:** Yen-Shan Chen, Joseph D. Racca, Michael A. Weiss

**Affiliations:** Department of Biochemistry and Molecular Biology, Indiana University School of Medicine, Indianapolis, IN, United States

**Keywords:** testis determination, gonadogenesis, organogenesis, protein-DNA recognition, infertility

## Abstract

Male sex determination in mammals is initiated by SRY, a Y-encoded transcription factor. The protein contains a high-mobility-group (HMG) box mediating sequence-specific DNA bending. Mutations causing XY gonadal dysgenesis (Swyer syndrome) cluster in the box and ordinarily arise *de novo*. Rare inherited variants lead to male development in one genetic background (the father) but not another (his sterile XY daughter). *De novo* and inherited mutations occur at an invariant Tyr adjoining the motif’s basic tail (box position 72; Y127 in SRY). In SRY-responsive cell lines CH34 and LNCaP, *de novo* mutations Y127H and Y127C reduced SRY activity (as assessed by transcriptional activation of principal target gene *Sox9*) by 5- and 8-fold, respectively. Whereas Y127H impaired testis-specific enhancer assembly, Y127C caused accelerated proteasomal proteolysis; activity was in part rescued by proteasome inhibition. Inherited variant Y127F was better tolerated: its expression was unperturbed, and activity was reduced by only twofold, a threshold similar to other inherited variants. Biochemical studies of wild-type (WT) and variant HMG boxes demonstrated similar specific DNA affinities (within a twofold range), with only subtle differences in sharp DNA bending as probed by permutation gel electrophoresis and fluorescence resonance-energy transfer (FRET); thermodynamic stabilities of the free boxes were essentially identical. Such modest perturbations are within the range of species variation. Whereas our cell-based findings rationalize the *de novo* genotype-phenotype relationships, a molecular understanding of inherited mutation Y127F remains elusive. Our companion study uncovers cryptic biophysical perturbations suggesting that the *para*-OH group of Y127 anchors a novel water-mediated DNA clamp.

## Introduction

**Figure 1:**
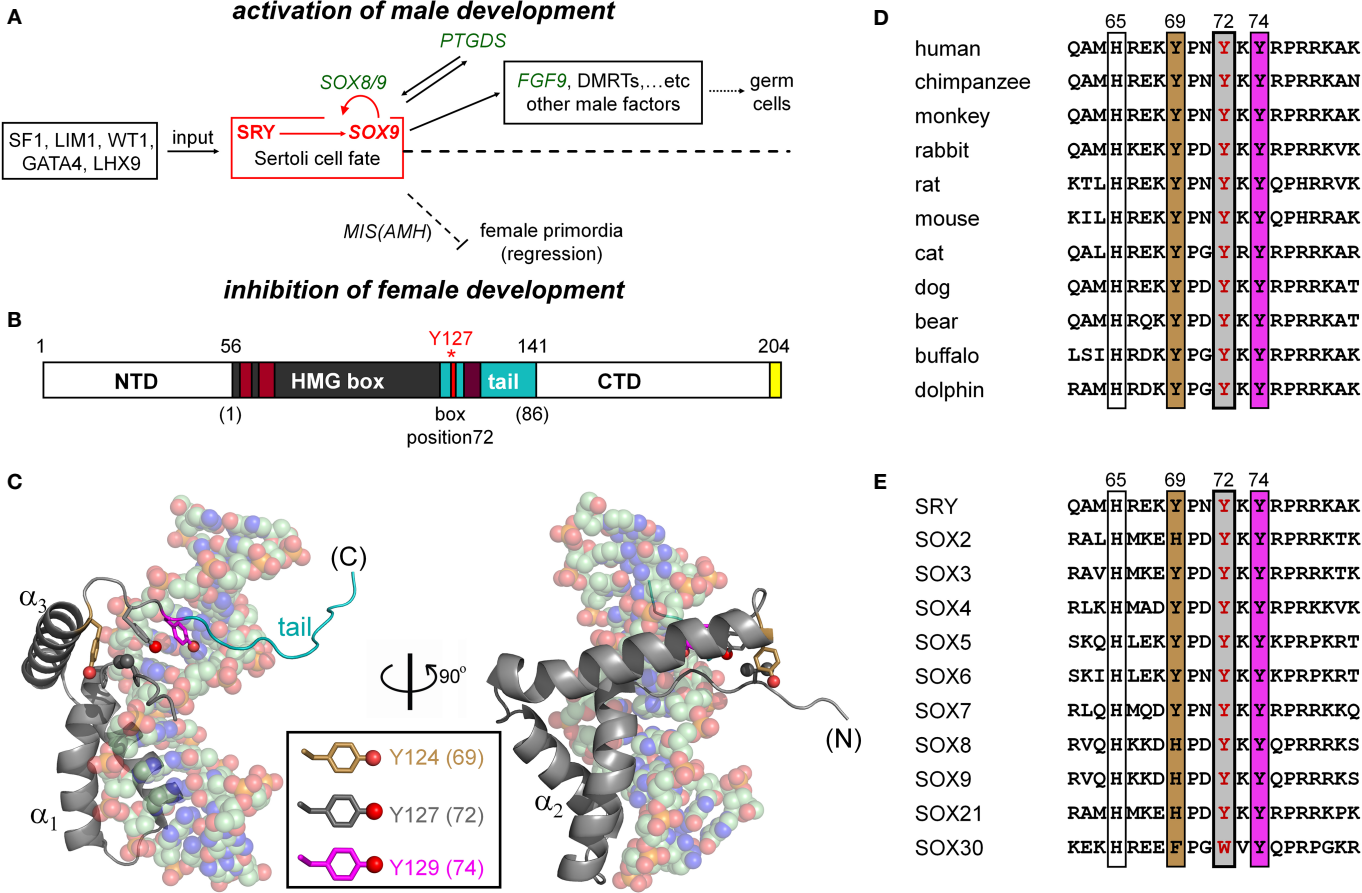
SRY-regulated gene-regulatory network (GRN) and structure of SRY HMG box. **(A)** SRY-dependent testis determination pathway. Red box highlights central SRY→SOX9 step (1, 2). Genetic inputs are at left and outputs at right. To effect gonadal-ridge differentiation, SOX9 activates a male-specific GRN, in turn directing Müllerian-duct regression (dashed ⊥; Müllerian inhibiting substance MIS; also designated anti-Müllerian hormone (AMH)) (3) while inhibiting the granulosa-cell fate (1, 3). **(B)** SRY domain organization with HMG box in gray. The box’s basic tail (light blue) comprises residues 56-141. The extreme C terminus of SRY contains a PDZ-binding motif (yellow), proposed to interact with SIP-1 (4). Above bar are indicated residue numbers in intact SRY; red asterisk highlights Y127 (box position (5); see consensus numbering scheme below bar. Nuclear localization signals N-NLS and C-NLS are respectively highlighted in light- and dark brown (6). **(C)** Solution structure of SRY box-DNA complex (Protein Databank entry 1J46) (7). The HMG box (ribbon; basic tail in light blue) docks within minor groove of bent DNA site (space-filling). The domain contains major- and minor wings, the latter comprises the N-terminal β-strand, the remainder of α-helix 3, and C-terminal tail. The minor wing is stabilized by an “aromatic box” in a mini-core containing the side chains of V60, H120, Y124, and Y127, extended by Y129 at tail-DNA interface (respective box positions 5, 65, 69, 72 and 74; parentheses). The three Tyr side chains (inset color code; residues) exhibit distinct structural environments. For clarity, the view at right reflects a 90° rotation about the vertical axis. **(D)** Sequence alignment among selected mammalian orthologs highlighting box residues 62-81. Conserved residues at positions 69, 72, and 74 are outlined. Gray-shaded box indicates position of the clinical variants characterized in the present study (Y127 in intact human SRY); filling colors as in **(C)**. **(E)** Corresponding alignment of human SOX homologs (8).

The male phenotype in therian mammals is determined by Sry^1^ (designated SRY in primates), an architectural transcription factor encoded by the pseudo-autosomal region of the Y chromosome (**Figure 1A**) (9, 10). In late embryogenesis, SRY initiates testicular differentiation of the bipotential gonadal ridge (10, 11). A downstream gene-regulatory network (GRN) has been investigated in mouse models (12–15), identifying autosomal gene *Sox9* as the principal target of Sry in embryonic pre-Sertoli cells (15). Sry binds to specific regulatory DNA sites in the upstream region of *Sox9* (or human *SOX9*), functioning as testis-specific enhancers (designated *TES* and *Enh13*) (16–19). Whereas *Sry/SRY* represents an evolutionary innovation in therian mammals, *Sox9* is broadly conserved among vertebrate sex-determining (VSD) pathways: an example of such pathways “growing backwards” in evolution (20, 21).

Sox9 (SOX9 in primate nomenclature) is an architectural transcription factor (22–24) and a member of the general class of eukaryotic SOX factors defined by an Sry-related high-mobility-group (HMG) box (25), a conserved motif of DNA binding and bending (**Figure 1B**) (26). Following its transcriptional activation by Sry in the embryonic gonadal ridge (red box in **Figure 1A**), a Sox9-dependent GRN orchestrates programs of cell-cell communication, migration, and differentiation leading to testis formation (**Figure 1A**; upper regulation pathway). Mutations at successive levels of this pathway are broadly associated with disorders of sexual development (DSD). Endocrine functions of the fetal testis, once formed, direct subsequent somatic virilization (*via* secretion of testosterone) with regression of the female anlägen (*via* glycohormone Müllerian Inhibiting Substance [MIS/AMH]) as outlined in **Figure 1A** (lower pathway). Genotype-phenotype relationships in DSD reflect the molecular logic of this pathway (15, 27).

Evidence for the critical role of SRY in human testis determination was provided by the discovery of diverse clinical mutations leading to 46, XY gonadal dysgenesis with female somatic phenotype (Swyer Syndrome) (15); for review, see (28)). Such mutations usually occur *de novo* in paternal spermatogenesis. Swyer variants exhibit a broad range of biochemical and biophysical perturbations (15, 27). Whereas *de novo* (sporadic) mutations in SRY are typically associated with marked impairment of specific DNA binding, a distinct class is inherited (29–31). The latter mutations are by definition compatible with alternative developmental outcomes: testicular differentiation leading to virilization (fertile 46, XY father) or nascent ovarian differentiation leading to gonadal dysgenesis with female somatic phenotype (sterile 46, XY daughter) (32–34). Inherited mutations in SRY thus provide “experiments of nature” that define mechanistic boundaries of genetic function. These boundaries may pertain to one or more biochemical activities, such as specific DNA binding, nucleocytoplasmic shuttling, post-translational modification, or proteasomal degradation (30, 31). Swyer mutations cluster in (but are not restricted to) the SRY HMG box (15, 27). Mutations in the HMG box of SOX9 can also cause XY sex reversal (22).

The sequence-specific HMG box is an L-shaped domain containing an N-terminal β-strand, three α-helices (α_1_, α_2,_ and α_3_), and C-terminal basic tail (**Figure 1C**). Packing of the β-strand against the C-terminal portion of α_3_ defines the *minor wing* in the HMG box, whereas α_1_, α_2,_ and the N-terminal portion of α_3_ comprise the *major wing*. Unlike structure-specific homologs (such as the boxes of HMG-1 and HMG-D (35, 36), which are generally well ordered in the absence of DNA (35, 37, 38), SRY’s minor wing is partly disordered: N- and C-terminal strands are unfolded, and the major wing is molten (7). DNA recognition is mediated within an expanded DNA minor groove; such binding in turn stabilizes the canonical L-shaped domain structure. The specific protein-DNA interface is remarkable for “wedge insertion” of nonpolar side chains (HMG-box positions 9, 12, 13, and 43; residues 64, 67, 68, and 98 in human SRY) in addition to canonical hydrogen bonds and salt bridges (39, 40). Specific contacts in the SRY-DNA complex are conserved within the homologous SOX co-crystal structures (41, 42). The SRY/SOX-DNA interface is extended by a C-terminal basic tail (blue in **Figure 1B**), which remains in the minor groove (7) to augment the kinetic stability of the bent complex (43).

**Figure 2:**
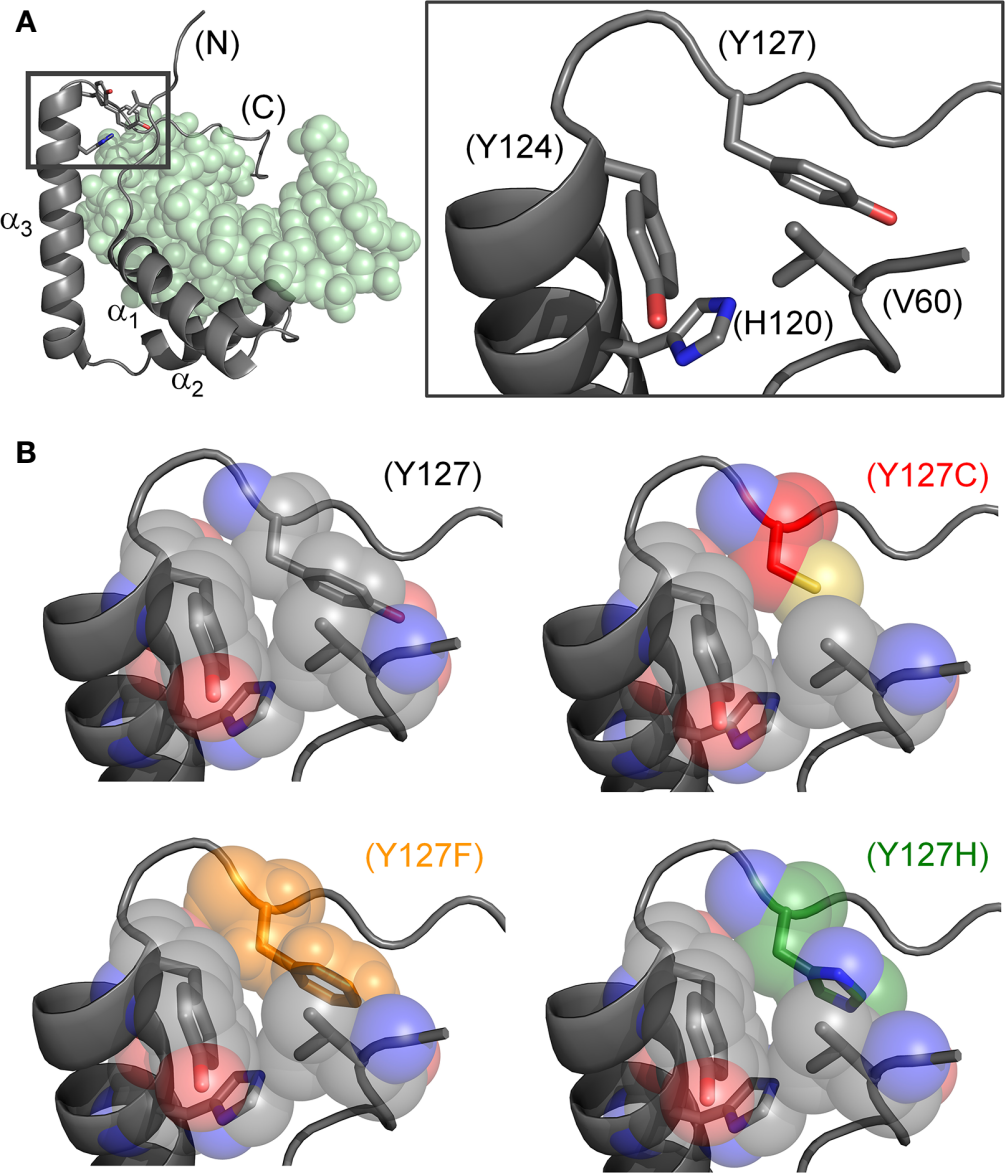
Structural models of wild-type and mutant side chains. **(A)** L-shaped SRY HMG box (α-helices labeled α_1-3_) bound to a DNA target site (light green spheres; PDB entry 1J46) (7). Shown at right is an expanded view of boxed region depicting minor-wing mini-core (full-length numbering scheme). **(B)** Rigid-body substitution of mutant side chains at box position 72 (sticks) in an otherwise transparent CPK model. Models of the three clinical mutants (34, 44, 45): Y127C in red, Y127F in orange and Y127H in green.

The present study has focused on three Swyer mutations at a conserved site at the junction between helix α_3_ and the basic tail: box position 72 and residue 127 in human SRY. This junction is conserved among Sry orthologs (**Figure 1D**) and SOX homologs (**Figure 1E**). Two of the mutations occurred *de novo* (Y127C and Y127H) whereas the third was inherited (Y127F). The wild-type (WT) side chain is of dual structural interest. On the one hand, one face of the aromatic side chain buttresses a DNA-binding surface (**Figure 2**) *via* a cluster of aromatic residues engaging V60 (box position 5): H120, Y124, and Y127 (respective box positions 65, 69, and 72). On the other hand, the *para*-hydroxyl group of Y127 faces the DNA phosphodiester backbone, but is too distant for direct engagement (6.7 Å from the nearest DNA atom; a non-bridging phosphodiester oxygen atom) (7). Whereas the *de novo* mutations would be expected to perturb the aromatic cluster—in turn perturbing an overlying DNA-binding surface—how and to what extent the inherited Y127F variant impairs SRY function poses an intriguing question. Although Tyr and Phe are similar aromatic amino acids and often interchangeable among homologous proteins, a previous study has reported that Y172F blocks detectable specific DNA binding by the variant SRY domain (34). If valid, this finding would pose a seeming paradox: how might the phenotype of the fertile 46, XY father be rationalized?

The present study sought to resolve this issue through comparative cell-based and biochemical studies of the three Swyer mutations (Y127C, Y127H, and Y127F). Whereas the two *de novo* mutations were found to markedly impair SRY function, the effects of Y127F are modest: indeed, substantial gene-regulatory function was retained in cell-based models of SRY function (30, 31) in accordance with its inheritance. Only subtle changes were observed in the variant domain’s thermodynamic stability, specific DNA affinity, and induced DNA bend angle. Based on homologous SOX-DNA crystal structures (41, 42, 46), we envision that the Y127 *para*-OH group anchors a water molecule at the protein-DNA interface and propose that the loss of a single water-mediated hydrogen bond attenuates the male switch at the edge of ambiguity. Our companion study in this issue (47) provides biophysical evidence that removal of the Y127 *para*-OH group “unclamps” the sharply bent protein-DNA complex to enhance long-range conformational fluctuations and accelerate its dissociation.

Together, our studies uncovered an otherwise cryptic contribution of a conserved aromatic residue to the function of human testis-determining factor SRY. This residue’s structure-activity relationships are likely to extend to the SOX family of architectural TFs (26) and more broadly to a metazoan superfamily of sequence-specific DNA-bending domains (35). Molecular mechanisms of a rare syndrome—Swyer syndrome as a probe of human genetic variation—may thus illuminate general principles of developmental gene regulation and its evolution.

## Materials and Methods

### Mammalian Plasmids

Plasmids expressing wild-type human SRY, clinical mutations, and controls were constructed by polymerase chain reaction (PCR) (30) from template WT human SRY gene. The cloning site encoded a hemagglutinin (HA) tag in triplicate to enable Western blotting (WB) and chromatin immunoprecipitation (ChIP). Mutations of SRY were introduced using PCR and QuikChange™ Multi Site-Directed Mutagenesis Kit (Stratagene). Constructions were verified by DNA sequencing.

### Cell Culture

Rodent CH34 cells (kindly provided by Dr. P.K. Donahoe, Massachusetts General Hospital) (30, 48) were cultured in regular DMEM medium containing 5% fetal bovine serum (FBS) at 37°C under 5% CO_2_. Human LNCaP cells (ATCC^®^ CRL-7002™ and ATCC^®^ CRL-1740™, respectively) were obtained from American Type Culture Collection (ATCC) and cultured in Dulbecco’s Modified Eagle Medium (DMEM) medium containing 10% FBS in 5% CO2 atmosphere.

### Transient Transfections

Transfections were performed using Lipofectamine 3000 as described by the vendor (Invitrogen). After 8 h in an improved Minimal Essential Medium (Opti-MEM; ThermoFisher), cells were recovered using fresh culture medium with FBS. Transfection efficiencies were determined by the ratio of green-fluorescent protein (GFP) positive cells to untransfected cells following co-transfection with pCMX-SRY and pCMX-GFP in equal amounts (30). Subcellular localization was visualized by immunostaining 24-h post transfection following treatment with 0.01% trypsin (Invitrogen) and plating on 12-mm cover slips. SRY expression was monitored by WB *via* its triplicate HA tag.

### Chromatin Immunoprecipitation

Cells were transfected with plasmids encoding epitope-tagged WT or variant SRY under 1X conditions (undiluted by the parent plasmid). Recovered cells were cross-linked in wells by formaldehyde, collected, and lysed after quenching the cross-linking reaction. Lysates were sonicated to generate 300-400-bp fragments and immunoprecipitated with anti-HA antiserum (Sigma) containing a Protein A slurry (Cell Signaling). A non-specific antiserum (control IgG; Santa Cruz) served as non-specific control. PCR and quantitative reverse-transcriptase-PCR (qPCR) protocols were as described (31). The pairs of forward (F) and reverse (R) DNA oligonucleotide primers employed to probe embryonic testis-specific enhancer elements (*TESCO* (16); mouse and human *Enh13* (17–19)).

### Western Blot

24-h post transient transfection, cells were split evenly into 6-well plates and treated with cycloheximide to a final concentration of 20 μg/ml in DMEM for the indicated times; cells were then lysed by RIPA buffer (Cell Signaling Technology). Protein concentrations were measured by BSA assay (ThermoFisher); cell lysates were subjected to 4%-20% SDS-PAGE and WB using anti-HA antiserum (Sigma-Aldrich) at a dilution ratio of 1:5000; α-tubulin antiserum provided a loading control. For phosphorylation analysis, HA-tagged SRY variants were immunoprecipitated with rabbit polyclonal anti-phosphoserine antiserum (Abcam). WB following 4%-20% SDS-PAGE employed HRP-conjugated anti-HA antibody (Roche). Quantification was performed by Image J software.

### Transcriptional Activation Assay

Following transient transfection (above), SRY-mediated transcriptional activation of SOX9/Sox9 was measured in triplicate by qPCR as described (29). In the transient transfections, the expression plasmid encoding HA‐tagged WT SRY or an HA‐tagged variant SRY was diluted 1:50 with the empty parent plasmid to reduce protein expression to the physiological range [ca. 103-104 protein molecules/cell (30)]. The expression levels were verified by WB. After the transient transfection, cellular RNA was extracted and converted to cDNA by using the vendor’s protocol (Bio‐Rad). SRY‐mediated transcriptional activation was probed by *SOX9/Sox9* mRNA as readouts. Primer sequences for all of the tested genes were applied as described (31, 55). An internal control was the specific 5′‐TATAADNA‐binding subunit of TFIID. Data analysis included three technical replicates of each of three biological replicates. Detail is provided as **Supplemental Information**.

### Cycloheximide-Chase Assay

Post-transfection cells were split evenly into 6-well plates and treated with translation inhibitor cycloheximide (final concentration is 20 mg/mL) in the regular medium for indicated times; cells were then lysed using mammalian lysis buffer (Hoffmann LaRoche). After normalization of total protein concentration by BCA assay (ThermoFisher), lysates were subjected to 4%-20% SDS-PAGE and Western blot using anti-HA antiserum (Sigma-Aldrich) at a dilution ratio of 1:5000 with αtubulin as a loading control. Quantification was performed by Image J program.

### SRY-DNA Permutation Gel Electrophoresis

Six DNA fragments (150 bp each) containing an SRY-binding site (5′-ATTGTT-3′ and complement) were PCR-amplified from a plasmid previously described (30) such that the binding site was at varying distances from the 5′-end (leading to variation in “flexure displacement”). Each fragment (10 nm) was complexed with the WT or variant HMG box (20 nm). Gels were purchased from Bio-Rad, equilibrated, and resolved in 0.5× Tris borate/EDTA buffer (TBE). Protein-DNA complexes were visualized using SYBR Green stain (Life Technologies).

### Immunocytochemistry

Transfected cells were plated evenly on 12-mm cover slips, fixed with 3% para-formaldehyde in PBS buffer solution, and then treated with permeability buffer solution (PBS containing 10% goat serum and 1% Triton X-100; Sigma-Aldrich) as described (30). SRY variants were probed using FITC-conjugated anti-HA antibody (Santa Cruz). cells were visualized by fluorescent microscopy; DAPI stained the nucleus. Nuclear localization was evaluated by the ratio of cells expressing SRY in the nucleus to the total number of GFP-positive cells and further analyzed by the subcellular fractionation assay (30).

### Inducible Tet-*On* SRY Expression System

DNA segments encoding WT SRY or variants were ligated into pTet-IRES-EGFP-SRY plasmid (parent plasmid purchased from Addgene (# 64238) (56)). Respective plasmids and their parent were purified, validated (56), and subjected to viral particle assembly 293FT cells as described by the vendor; this system exploits pLenti CMV rtTA3 Blast (for reverse tetracycline-controlled trans-activator expression). Equal volumes of the latter and pTet-IRES-EGFP-SRY viral particles were then mixed for transduction in 6-well plates containing *ca*. 1 million mammalian CH34 or LNCaP cells. Expression of the WT or variant epitope-tagged SRY was induced by the addition of 100 ng/mL doxycycline to a maximum concentration of 800 ng/mL (Sigma Aldrich). Titration of doxycycline and its associated SRY expression levels is described in the **Supplemental Discussion** and **Figure S1**.

### CD-Based Protein Stability

Thermodynamic stabilities of WT and variant HMG domains in 10 mM potassium phosphate buffer, 140 KCl (pH 7.4) at 25°C were determined using guanidine-hydrochloride titrations, monitored by CD at α-helix-sensitive wavelength 222 nm (57). Using non-linear least-squares regression, relationships between ellipticity and guanidine concentration were fit to a two-state model (58):




$$\text{Θ}\left(C\right)=\frac{{\text{Θ}}_{A}+{\text{Θ}}_{B}e\left(-\Delta G-mc\right)/RT}{1+e^{\left(-\Delta G-mc\right)}/RT}$$

where Δ*G*Δ*G* is the Gibbs free energy of unfolding, *cc* is guanidine concentration, *RR* is the ideal gas constant, *TT* is temperature, and *Ɵ_A_Ɵ_A_* and *Ɵ_B_Ɵ_B_* are baseline ellipticity values reflecting the folded and unfolded states, respectively. Baseline ellipticities were calculated *via* simultaneous fitting of linear equations *Θ*_*A*_(*C*)=*Θ*_*A*_+*m*_*A*_ *c* and *Θ*_*B*_(*C*)=*Θ*_*B*_+*m*_*B*_ *c* (59). Thermal unfolding of the free domains and equimolar protein-DNA complexes (25 μm in standard buffer) were monitored using a 15-bp DNA duplex (5′-TCGGTGATTGTTCAG-3′ and complement; consensus SRY target sequence underlined); CD measurements were acquired from 4-90°C at 0.5°C increments at 222 nm.

### Fluorescence Resonance Energy Transfer

FRET studies of protein-directed DNA bending employed a 15-bp DNA duplex (sequence 5’-TCGGTGATTGTTCAG-3’ (“upper strand”) and complement; consensus target site underlined. Use of a 15-bp DNA site restricts protein binding to the 1:1 high-affinity complex (29). To provide a fluorescent donor, the upper strand was extended at its 5’ terminus by 6-carboxyfluorescein (6-FAM); the dye was flexibly linked to the DNA through a hexanyl linker. To provide a partner acceptor, the lower strand was extended at its 5’-end by tetramethylrhodamine (TAMRA), also *via* a hexanyl linker. Previous photophysical control studies demonstrated the flexibility of the fluorophores (43, 60). Labeled DNA strands were purchased from Oligos, Etc., Inc. (Wilsonville, OR). In these assays the DNA site was made 5 μM at pH 8.4 in 10 mM potassium phosphate, 10 mM Tris-HCl, 140 mM KCl, 1 mM ethylenediaminetetraacetic acid (EDTA), and 1 mM dithiothreitol (“FRET buffer”).

### FRET-Based Protein-DNA Titrations

Steady-state FRET was employed to determine protein-DNA dissociation constants (*K*_d_); the DNA site was as above. Measurements were made in FRET buffer at 15°C and. Varying concentrations of the wild-type or variant SRY domain were titrated at a constant DNA concentration of 25 nM. Emission spectra were recorded from 500-650 nm following excitation at 490 nm. Estimates of *K*_d_ were determined by plotting the change in fluorescence intensity at 520 nm against total protein concentration. Data were fit with a single-site ligand-binding model (Equation 1) as described (61) using *Origin 8.0* software (OriginLab Corp., Northampton, MA).


(1) $$\Delta F=\;\Delta F_{\text{o}}\left\{0.5\left(1+S/D_{\text{o}}+K_{\text{d}}/D_{\text{o}}\right)-{\left[0.25{\left(1+S/D_{\text{o}}+K_{\text{d}}/D_{\text{o}}\right)}^{2}-S/D_{\text{o}}\right]}^{0.5}\right\}$$

In this formalism Δ*F* is the change in donor fluorescence observed on addition of the SRY domain relative to the baseline DNA fluorescence; Δ*F*_0_ is the maximum fluorescence change obtained in a 1:1 protein-DNA complex; *K*_d_ is the dissociation constant; *D*_0_ is the concentration of DNA (25 nM); and S is the concentration of SRY domain.

## Results

Two SRY-responsive mammalian cell lines (embryonic rat pre-Sertoli XY CH34 (48)) and human prostate-cancer-derived LNCaP (62, 63)) provided models for functional analyses of wild-type (WT) and mutant SRYs in culture (see **Supplemental Discussion, Supplemental Figures S1–S3**, and **Table S1**) (30, 63). The independent origins of these cell lines were intended to mitigate possible line-specific or species-specific effects unrelated to their shared SRY responsiveness. Following expression of WT or variant SRY by transient transfection (with plasmid dilution to control expression level (30)), SRY-directed transcriptional activation of *Sox9*/*SOX9* was evaluated in their respective native chromosomal contexts. Employing qPCR, this assay measured the time-dependent accumulation of mRNAs encoded by downstream genes in the SRY-*SOX9* GRN (10). Level of expression of the transfected SRYs was set in the physiological range (10^3^-10^4^ mean molecules per cell) by an empty-plasmid dilution or Tet-on expression protocols (see **Supplemental Discussion**, **Supplemental Figure S4** and **Supplemental Table S2**) (30, 64). Because mutations may in principle affect expression level at a given plasmid-dilution ratio or Tet concentration, functional studies were repeated on chemical proteasome inhibition to equalize WT or variant SRY expression (below).

**Figure 3:**
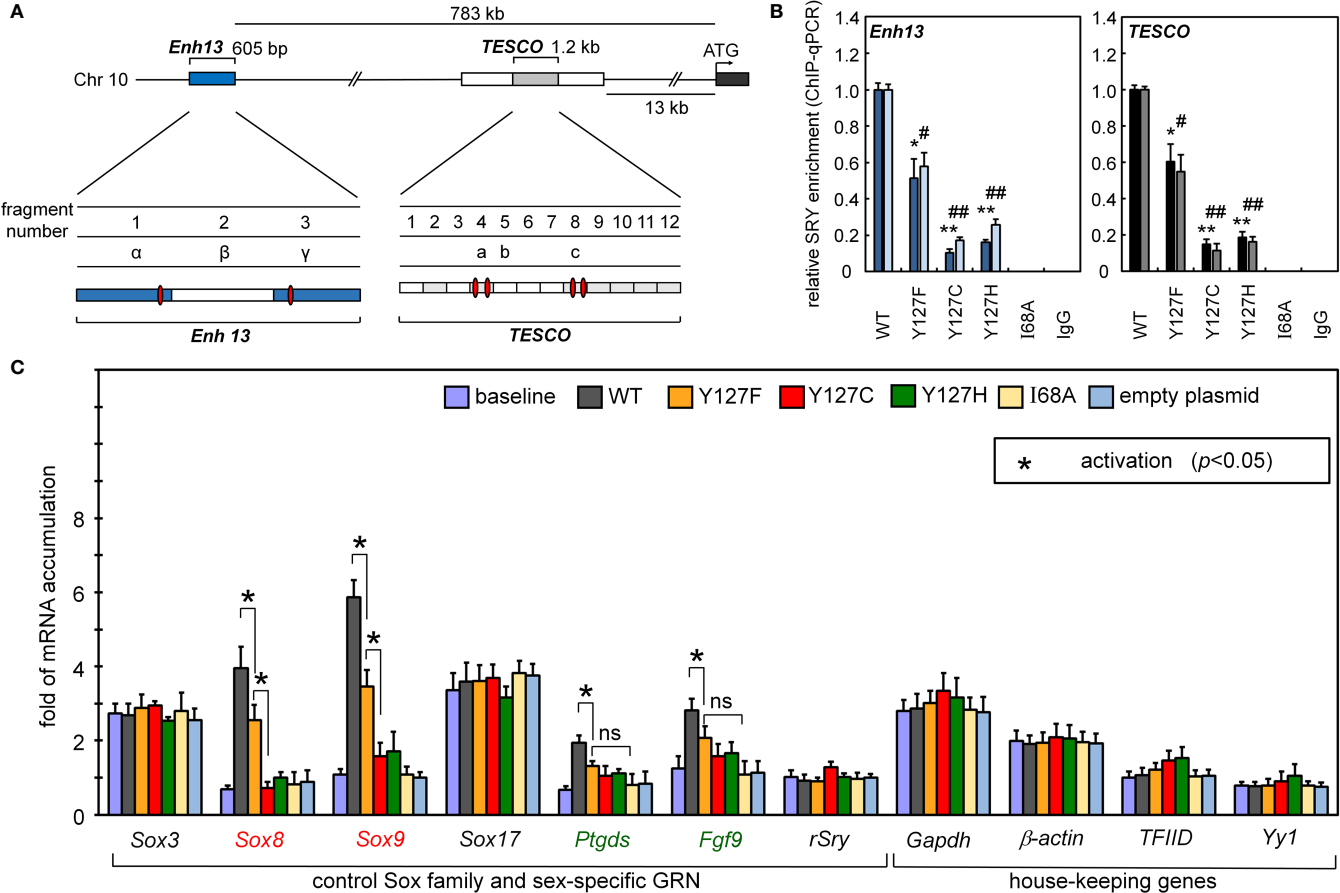
Gene-regulatory activities of SRY variants. **(A)** During differentiation of the embryonic gonadal ridge, rodent and human *Sox9/SOX9* genes are each regulated by upstream testis-specific enhancer elements: Enh13 (17) (blue) and TESCO (16) (light gray). Potential SRY-binding sites in Enh13 are present in fragments 1 and 3 (related to primer sets α and β) and in TESCO fragments 4 and 8 (primer sets a and c). **(B)** Effects of mutations in SRY HMG box on enhancer occupancies. Respective SRY-Enh13 (left) and SRY-TESCO (right) occupancies were probed and quantified by ChIP-qPCR (16, 17) as indicated by respective histograms. Results were normalized relative to WT SRY (at left in each panel). Respective control lanes I68A indicate an inactive SRY (39); IgG lanes indicate nonspecific control (* and #), (** and ##) indicate statistical significance at respective levels p<0.05 and <0.01. **(C)** Transcriptional survey of selected genes pertinent to male development (30). Surveyed mRNAs include sex determination specific genes *Sox8*, *Sox9*, *Ptgds*, *Fgf9*, and original endogenous rat Sry; controls were provided by housekeeping genes (30). In each case baseline mRNA abundances were determined in non-transfected cells. Inset box, statistical significance at the level p<0.05 (Wilcox test) for SRY-dependent transcriptional activation (*).

Occupancies of SRY binding sites within corresponding far-upstream testis-specific enhancer elements of *Sox9/SOX9* (17) were probed by ChIP (17). The protein binds within the core elements of two testis-specific enhancer elements (designated *TESCO* and *Enh13*, respectively; **Figure 3A**); results are summarized in **Figure 3B**. The three Swyer substitutions were evaluated at SRY position 127 (an inherited mutation Y127F, *de novo* clinical variants Y127C and Y127H), and negative controls by expression of I68A (box position 13), in which a known clinical mutation at the “cantilever” position of the SRY HMG box markedly impairs its specific DNA binding (65). In both cell lines, the SRY variants at position 127 exhibited, to varying extents, reduced *TESCO*/*Enh13* enhancer occupancies. Whereas inherited Y127F SRY preserved *ca*. 60% of occupancy relative to WT (as quantified by ChIP-qPCR; **Figure 3B**), *de novo* clinical variant Y127C and Y127H exhibited ~20% occupancy in both cell models (**Figure 3B**). As expected, control mutation I68A blocked detectable binding of the variant SRY at either the *TESCO* or *Enh13* enhancers.

Reducing the formation of testis-specific enhanceosomes directly impairs *Sox9* gene activation (**Figure 3C**). Further, because *SOX9* is the principal factor target of SRY, and SOX9 in turn activates the downstream GRN (**Figure 1A**, sequential to or in synergy with SRY), specific upregulation of *Sox8* was reduced twofold on transient transfection of Y127F SRY (relative to WT) and more markedly by the two *de novo* mutations. Similarly, sequential activation of downstream genes *prostaglandin D2 synthetase* (*Ptgd2*) and *fibroblast growth factor 9* (*Fgf9*) was also reduced by all three mutations, whereas in control studies transient expression of SRY did not affect mRNA accumulation of non-sex-related Sox genes or unrelated housekeeping genes (**Figure 3C**).

**Figure 4:**
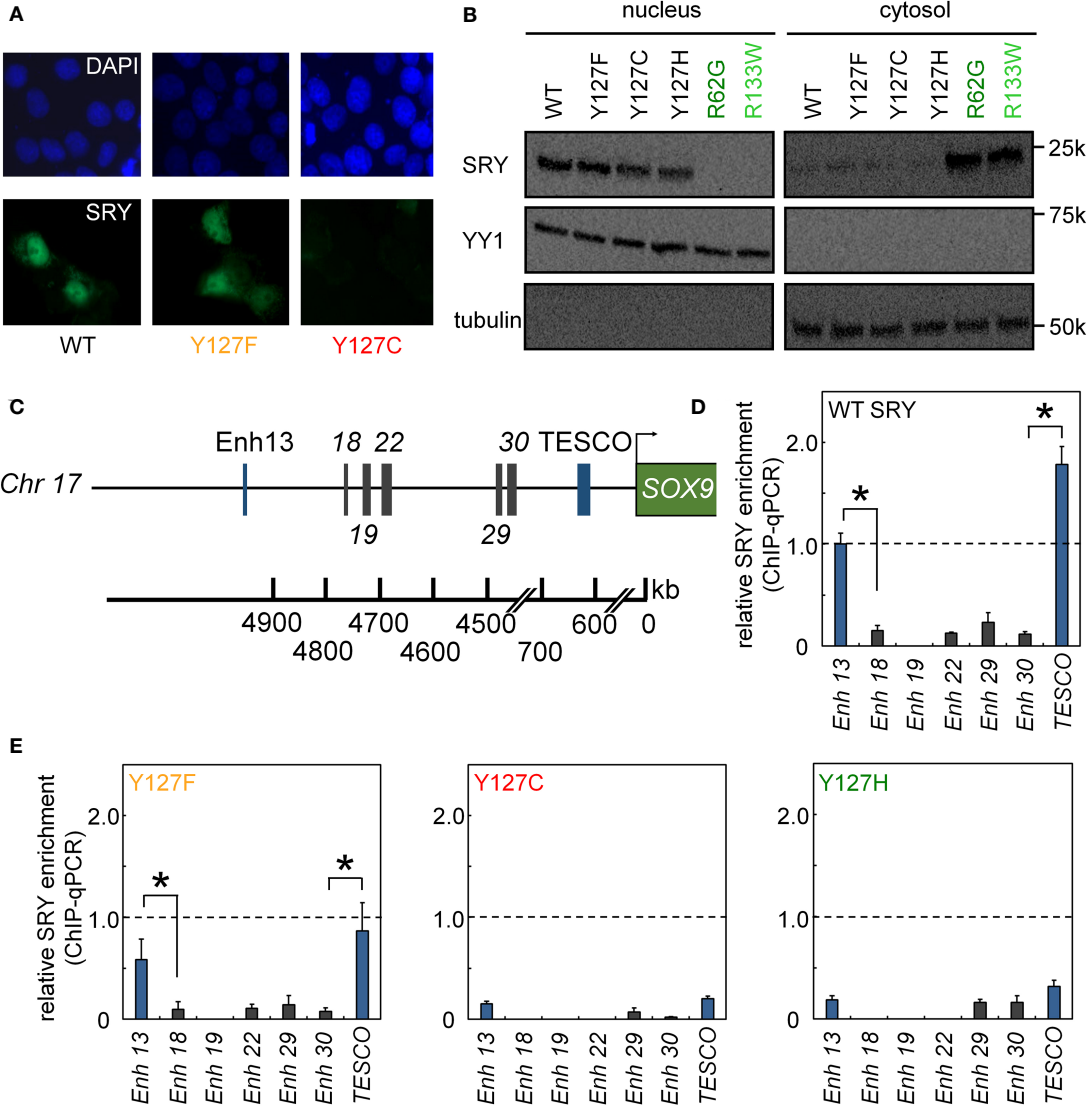
Nuclear localization and gene-regulatory activities of SRY variants. **(A)** Subcellular localization of epitope-tagged SRY constructs as analyzed by immunostaining (30): DAPI nuclear staining (upper row blue) and SRY (lower row green). WT and Y127F SRY accumulate primarily in nuclei but with underlying pancellular pattern (30). Y127C SRY exhibited decreased accumulation (see **Figure 6**). **(B)** Biochemical fractionation of SRY variants was analyzed by immunoblotting in cytoplasmic or nuclear fractions (30). Controls were provided by clinical variants R62G (defective N-NLS) R133W and R133W (defective C-NLS), as shown in dark- and light green, respectively. **(C–E)** Specific enhanceosome formation is perturbed by mutations. **(C)** *Sox9*/*SOX9* enhancers containing potential SRY binding motif are outlined in schematic form; code follows ref (17); DNA sequences are given as **Supplemental Information**. **(D, E)** ChIP-qPCR results delineate distribution of relative SRY occupancies as regulated by either WT SRY **(D)** and variants Y127F, Y127C, and Y127H **(E)**. Statistical significance at the level p<0.05 (Wilcox test) for SRY-dependent transcriptional activation (*).

We next analyzed subcellular localization. SRY contains two nuclear localization signals (NLS), one near the N terminus of the HMG box and the other near its C terminus (66–68). To test whether the mutations decreased the nuclear localization since position 127 is near the C-terminal NLS motif (**Figure 1B**), we tested its nuclear import by immunocytochemistry approach (**Figure 4A**) and cellular fractionation assay (**Figure 4B**). Immunostaining and subcellular fractionation studies (validated by respective nuclear and cytoplasmic markers YY1 and tubulin, respectively) demonstrated that the mutant SRYs retained predominantly nuclear localization, like WT (**Figures 4A, B**). In these assays, control Swyer mutations R62G (perturbing N-NLS) and R133W (C-NLS) impaired nuclear entry as expected. To test whether the mutations decreased testis-specific enhancer occupancies by enhancing off-target DNA binding in the same chromosomal regions, we analyzed potential flanking sites of ChIP occupancies in CH34 cells (**Figures 4C-E**). In addition to the two validated testis-specific enhancer elements in the *Sox9* gene, there are five additional chromosomal segments containing consensus or near-consensus SRY-binding DNA sequences (**Figure 4C**). Whereas WT SRY binds within *Enh13* and *TESCO* enhancers much more strongly than other potential DNA binding sites (**Figure 4D**), mutations perturbed enhancer occupancy without increasing off-target DNA binding (**Figure 4E**). These findings suggest that the mutations do not confer altered or relaxed DNA sequence specificity.

**Figure 5:**
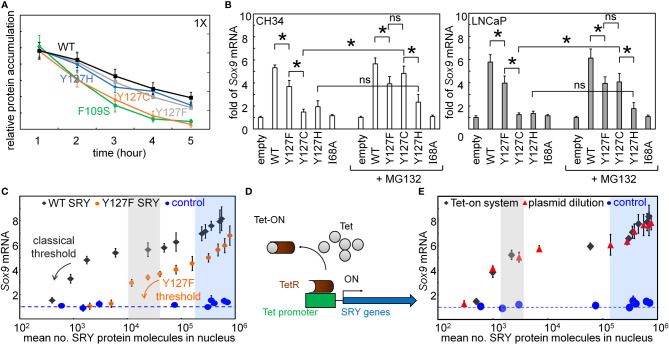
Assays of transcriptional activity. **(A)** Cycloheximide assay. Whereas Y127F and Y127H did not affect degradation (relative to WT), proteolysis was accelerated by Y127C [to an extent similar to that induced by clinical variant F109S (31)]. **(B)** Effects of chemical proteasome inhibition on gene-regulatory activities in rodent cell line CH34 [(48); left] and human cell line LNCaP [(62, 63); right]: fold-change in *Sox9*/*SOX9* gene expression in absence (left) or presence (right) of MG132. The latter alters activity of Y127C SRY, but not Y127F or Y127H SRY. Statistical analyses: “ns” indicates no significant difference, asterisks indicate p<0.05. **(C)** Relationship between WT SRY or Y127F SRY expression at the protein level (absolute units; molecules/cell) and fold-gene activation: (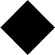) WT human SRY, (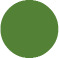) inherited variant Y127F SRY, and (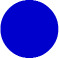) inactive I68A SRY control. Levels of WT, Y127F, and I68A SRY were controlled by the Tet-on system as in **(C)** Arrows indicate respective WT and Y127F thresholds protein concentrations (black and orange, respectively). **(D)** Schematic mechanisms of Tet-on system (70), which enables tuning of SRY expression in physiological range [10^3^-10^4^ protein molecules/cell (30)]. **(E)** Relationship between SRY/Sry expression at the protein level (absolute units; molecules/cell) and fold-gene activation: (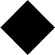) SRY expression regulated by Tet-on system, (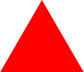) SRY expression controlled by encoded plasmids dilutions, and (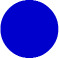) I68A SRY as inactive control. Shaded areas indicate TF thresholds (blue or gray), below which Sox9 mRNA abundance is attenuated and above which such expression is robust to changes in SRY/Sry concentration. Blue-shaded region also indicates marked TF over-expression. Shading and color code are as in **C**.

The cellular lifetime of SRY, its mean expression level, and rate of proteasomal degradation define additional biochemical properties affecting mean transcription-factor concentration and hence the extent of target gene activation (31, 69). We thus probed the proteolytic stabilities (**Figure 5A**). Cellular turnover of the expressed epitope-tagged SRY proteins was evaluated following treatment of cycloheximide, a chemical inhibitor of translation (**Figure 5A**). The Y127C variant was found to be more rapidly degraded than WT SRY or the other variants (**Figure 5A**). Such differential degradation could be circumvented through the addition of chemical proteasome-inhibitor MG132 to “rescue” expression of Y127C SRY. Such equalization of intracellular protein concentrations in large part rescued the gene-regulatory potency of Y127C SRY in the two cell lines (**Figure 5B**). No effect of MG132 was observed in the corresponding study of WT SRY or the other variants.

**Figure 6:**
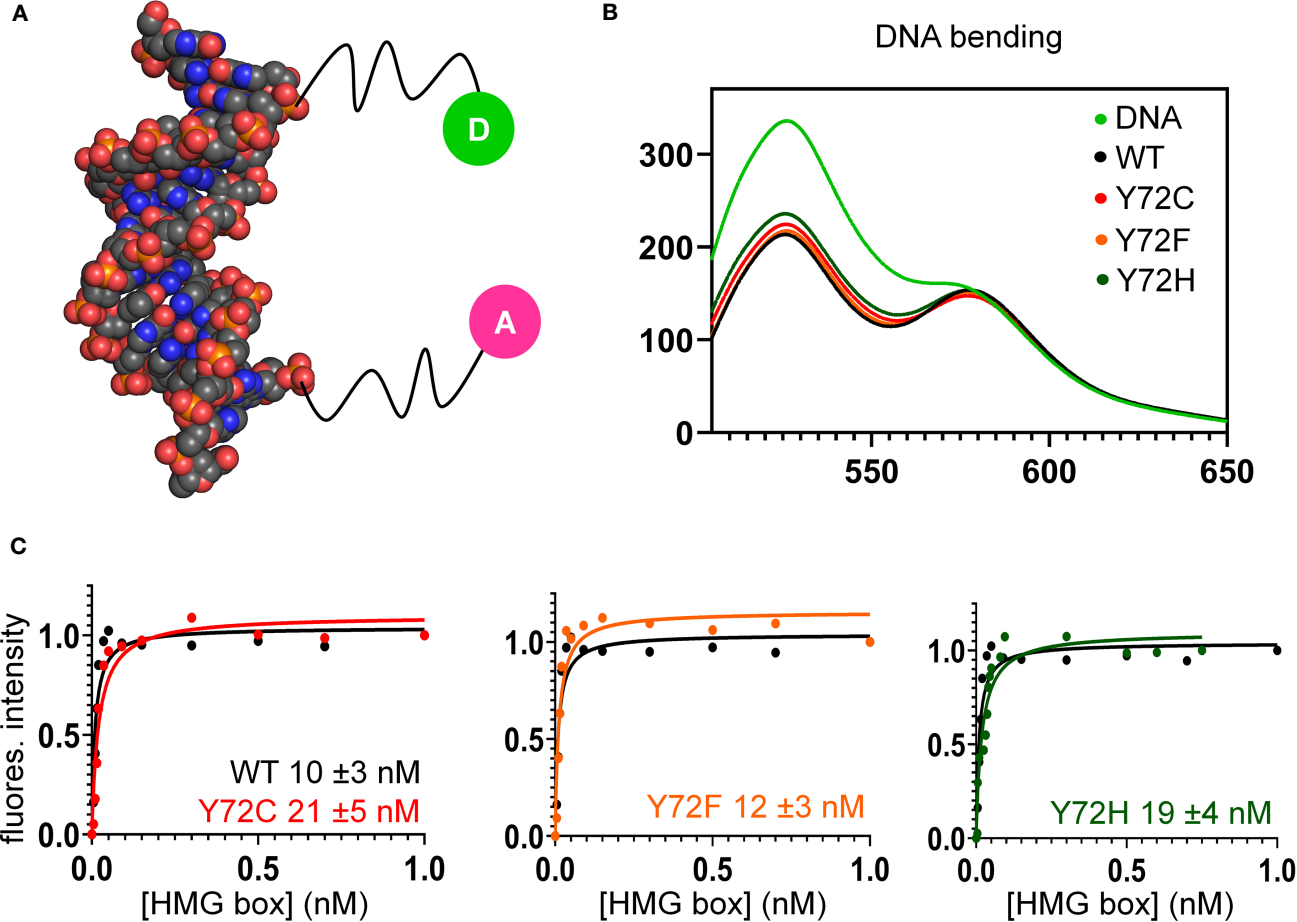
FRET-based equilibrium protein-DNA binding studies. **(A)** FRET probe: space-filling model of DNA (derived from Sox18 co-crystal structure; PDB entry 4Y60) modified at respective 5’-ends by fluorescent donor or acceptor, each *via* a 6-carbon flexible linker. The donor is fluorescein (green circle labeled “D”), and acceptor, *tetra*-methylrhodamine (TAMRA; fuchsia circle labeled “A”) as described (82). **(B)** Steady-state FRET spectra of WT and variant protein-DNA complexes. On protein-directed DNA bending, donor emission is reduced relative to an unbent protein-free DNA control (light green) due to FRET. **(C)** FRET-based equilibrium DNA binding studies of the WT and variant domains. In each titration WT data are plotted (black) relative to Y72F (left; orange), Y72C (center; red) and Y72H (right; green). Inferred equilibrium binding constants are inset. These values are robust to kinetic artifacts that can associated with gel mobility-shift assays (43).

Plasmid-dilution ratio provides a classical approach to control the intracellular concentration of SRY on transient transfection. Systematic exploration of plasmid-dilution ratios enabled the construction of a dose-response curve relating mean intracellular SRY concentration to fold-activation of the *Sox9* target gene (**Figure 5C**). This input-output relationship exhibits switch-like behavior: the toggle range below which *Sox9* expression declines (black arrow in **Figure 5C**) is 1-4 x 10^4^ mean protein molecules per cell (gray strip). Because WT and Y127F SRY exhibit different gene-regulatory properties on a per-molecule basis (above), their respective dose-response relationships fall on different curves (black and orange arrows in **Figure 5C**). Interestingly, Y127F SRY was found to be not equipotent to WT SRY even in the plateau region (from 10^5^-10^6^ mean protein molecules per cell), and its toggle range (arrow in **Figure 5E**) was shifted to the right in seeming agreement with the greater sensitivity of the mouse Sry-regulated intersexual development. No dose-response relationship was observed in control studies of inactive SRY variant I68A (blue circles in **Figure 5C**). The WT input-output relationship was replicated in a complementary Tet-*on* inducible system (shown in schematic form in **Figure 5D**; see also **Supplemental Figure S4**) as shown in **Figure 5E** (overlapping gray circles *vs.* plasmid-dilution-derived red circles). That two independent methods to control SRY expression levels in cell culture gave indistinguishable results suggests that this input-output relationship is an intrinsic feature of the SRY-*SOX9* regulatory axis.

Together, the above cell-based results indicated that de novo variants Y127C and Y127H are unable to activate downstream target gene *Sox9/SOX9*. Inherited allele Y127F by contrast retained partial gene-regulatory activity. To characterize the specific DNA-binding and bending properties of the variant HMG boxes in vitro, we next exploited a DNA-based FRET assay (**Figure 6A**): a protein-directed reduction in end-to-end DNA distance provided a signature of specific DNA binding and bending. This assay was based on differences in emission spectra reflecting donor intensity (**Table 1**, column 5). Spectra of WT and Y127F box-DNA complexes (respective black and orange traces in **Figure 6B**) essentially overlap, indicating similar FRET efficiencies. FRET efficiency in the Y127C box-DNA complex was slightly attenuated whereas that in the Y127H complex was the most attenuated. Specific DNA affinity was also determined by FRET titration (29). Remarkably, the variant boxes each retained high affinity for the consensus DNA target site (**Figure 6C** and **Table 1**, column 4), quantitatively similar to WT (**Figure 6C**, black traces). Specific DNA affinities and DNA bend angles are thus uncorrelated.

**Figure 7:**
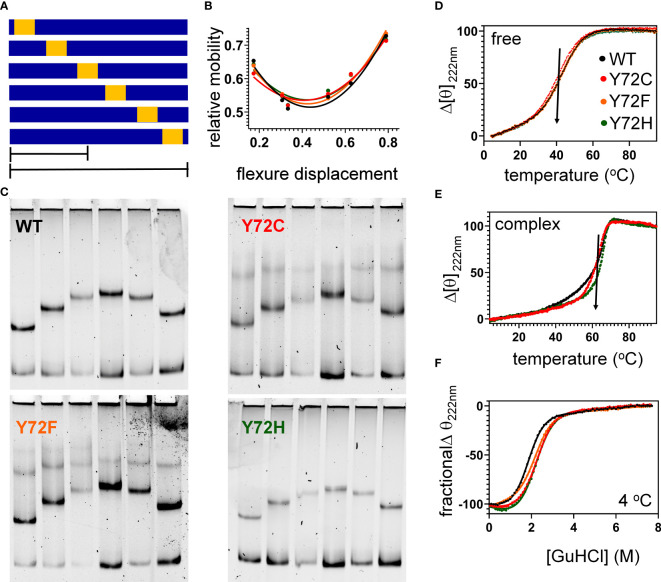
Protein-directed DNA bending. **(A)** Schematic representation of set of six DNA fragments used in permutation gel electrophoresis (PGE) (83). Golf region indicates position of SRY target site (core element 5’-ATTGTT-3’ and complement). Flexure displacement (designated by the two black lines) is the determined by dividing the number of base pairs from 5’-end to the center of the 5’-ATTGTT-3’ motif by total number of base pairs in the fragment (150 bp). **(B)** Relationship between flexure displacement (horizontal axis) and relative mobility for the WT or variant SRY domains (color code as inset in **D**). **(C)** Representative gels indicating that sharp DNA binding is retained for all variant domains with some attenuation in bend angle (see **Table 1**). **(D, E)** Thermal unfolding curves of free domains **(D)** and corresponding specific DNA complexes (**E**). Whereas the free domains indicate similar midpoint unfolding temperatures (T_m_) (near 40 °C; black arrow), the specific domain-DNA complexes are more resistant to thermal unfolding but exhibit different high-temperature behaviors. **(F)** Chemical denaturation studies of the free domains: CD-defined ellipticity at helix-sensitive wavelength 222 nm (vertical axis) was measured as a function of guanidine-HCl (horizontal axis); see **Table 1** for results of two-state thermodynamic modeling (58).

To further characterize protein-directed DNA bending, we employed permutation gel electrophoresis (PGE) to estimate bend angles (30). A consensus SRY target site was placed at varying distance from the 5’-end of a 150-bp DNA duplex (**Figure 7A**; target site in gold). Relative electrophoretic mobilities (**Figure 7B**) were analyzed as a function of flexure displacement (i.e., the distance of the DNA site from the 5’-end of the oligomer divided by 150 bp; **Figure 7C**). These estimates are qualitatively consistent with relative FRET efficiencies (**Table 1**, column 6). Although each variant retained sharp DNA bending, the FRET- and PGE-based results together thus demonstrated that the identity of residue 72 modulates the precise degree of bending. As expected (based on the unbound minor wing’s flexibility (29, 30), CD-based assays demonstrated that the free domains each retained nativelike thermal stability (**Figure 7D**) and thermodynamic stability (**Figure 7F** and **Table 1**, columns 7 and 8). In addition, each variant box-DNA complex was stably maintained at 37°C at a protein-DNA concentration of 25 mM (**Figure 7E**). These in vitro data do not correlate with relative rates of proteasomal degradation in a cellular milieu (above).

## Discussion

The interaction between the SRY HMG box and its specific DNA target site (core consensus sequence 5’-ATTGTT and complement (71)) is remarkable for bidirectional induced fit: (i) protein-directed DNA bending within a widened and underwound minor groove (72), (ii) DNA-directed folding of the minor wing of the HMG box extended by its basic tail (73). Such binding also stabilizes the major wing (31, 69). In the free box, the N-terminal segment is detached from helix α_3_; the tail is disordered. The minor wing’s mini-core stabilizes—and is stabilized by—an overlying protein-DNA interface. Critical to such bidirectional induced fit is the insertion of the aliphatic side chain of V60 (box position 5) into a cluster of aromatic side chains: H120, Y124 and Y127 (respective box positions 65, 69, and 72; **Figure 1C**). Paired proline residues C-terminal to α_3_ orient Y129 (box position 74) and successive basic residues to further stabilize the bent DNA complex. DSD mutations in these structural elements (variously perturbing mini-core packing, tail orientation, or direct side chain/DNA contacts) result in 46, XY gonadal dysgenesis with somatic sex reversal. These elements are invariant features of eukaryotic Sox boxes (8), including a junctional aromatic residue at box position 72 (Tyr or Trp; shaded gray rectangle in **Figures 1D, E**); corresponding clinical mutations have been identified in the HMG boxes of several SOX factors (*e.g*., SOX2, SOX4, SOX5, SOX9, SOX10, SOX11, SOX17, and SOX18 (5, 46, 74–77)) in association with diverse phenotypes.^2^ Such conservation suggests that the present structure-activity relationships, although probed only in SRY, will generalize to this broad family of architectural transcription factors (8).

Our study has focused on three clinical mutations at SRY residue 127 (consensus box position 72). Two occurred *de novo* (Y127C and Y72H): these presumably impair specific DNA-dependent folding of the minor wing through two mechanisms: loss of side-chain volume leading to a destabilizing cavity within the minor wing’s mini-core together with attenuation (Y127H) or loss (Y127C) of favorable aromatic-aromatic interactions. Y127C may also cause steric clash due to the larger atomic radius of sulfur relative to carbon at the γ side-chain position (**Figure 2**). Whereas nativelike domain-DNA affinities are maintained (column 4 in **Table 1**), the extent of DNA bending is reduced (column 6 in **Table 1**). Estimates of SRY-directed DNA bend angles, as inferred from PGE (**Figure 7**), yield ΔΔθ values of 6( ± 0.5)° (Y127H complex) and 9( ± 0.9)° (Y127C complex); these estimates are in qualitative accord with steady-state FRET spectra. Such attenuated DNA bending must represent a *transmitted effect* of the mutations as the WT side chain, and presumably also the variant side chains, is not in direct contact with the bent DNA site. These DNA bend angles each fall, however, within the range of WT species variation (60), so altered DNA bending is unlikely to be responsible for either the variant proteins’ loss of transcriptional activity in cell culture or associated DSD phenotypes (44, 45).

**Table 1:** Properties of WT and variant proteins.

HMG box^a^	fold *Sox9* activation^b^	*Sox9* activation (%)^c^	K_d_ (nM)^d^	FRET donor intensity^e^	DNA-bend angle^f^	ΔG_u_ ^g^ (kcal/mol)	C_mid_ ^h^ (M)
WT	5.6 ± 0.3	100	10 ± 3	63.5%	86° ± 2	2.8	1.8
Y72C	1.5 ± 0.3	12 ± 3	21 ± 5	66.8%	77° ± 6	3.0	2.2
Y72F	3.7 ± 0.5	61 ± 12	12 ± 3	64.7%	82° ± 6	3.0	2.2
Y72H	2.0 ± 0.5	22 ± 7	19 ± 4	70.1%	80° ± 5	2.8	2.1

a Residue numbers refer to consensus Box position; residue 72 corresponds to residue 127 in full-length human SRY.

b Fold-activation of Sox9 was analyzed following transient transfection (50X dilution) without chemical proteasomal inhibition. The number is normalized by the amount of endogenous Sox9 (set as 1).

c WT SRY at 50X dilution activates Sox9/SOX9 expression by 5.6 ± 0.3-fold activation relative to the empty-plasmid control. Relative Sox9 activation was calculated in relation to “net” differences in mRNA abundances between SRY-directed and basal levels of transcription.

d Affinities were determined at 15°C.

e Relative FRET donor intensities represent the ratio of the donor intensity of the bent DNA-protein complex compared to donor intensity in the free DNA probe. Errors are estimated to be ±0.2%.

f Bend angles were inferred from permutation gel electrophoresis at 4°C.

g Thermodynamic stabilities (± 0.1 kcal/mole) were extracted from chemical-denaturation assays at 4°C.

h Values indicate concentration of denaturant (± 0.01 M) wherein the domain is half-unfolded.

In embryonic pre-Sertoli cell line CH34 (40), Y127C SRY also underwent accelerated proteasomal degradation. Although the origins of such cellular turnover are not clear (the variant domain exhibits nativelike thermodynamic stability; column 7 in **Table 1**), restoration of native protein expression by chemical proteasome inhibition (histogram at right in **Figure 5**) partially rescued transcriptional activity (as monitored *via* autosomal target gene *Sox9*). Such partial rescue unmasks a subtle intrinsic defect in transcriptional activity. This modest perturbation (*ca*. twofold) stands in contrast to the more marked intrinsic defect in the transcriptional activity of Y127H SRY (*ca*. fivefold). These findings are counterintuitive as a nonpolar side chain (Cys) would in general be expected to be more perturbing than a weakly polar side chain (His) near a protein-DNA interface. To gain further insight into structure-activity relationships at box position 72, it would be of future interest to survey the specific enhancer-binding properties (in cells) and specific DNA-binding/bending properties (*in vitro*) of variant SRYs containing a diverse collection of substitutions at this site. The recent development of a CRISPR/Cas9-based mouse model of human SRY promises an opportunity to correlate such data with developmental outcomes in a mammal (55).

Of particular interest is inherited variant Y127F. This mutation, often observed as a conservative substitution within the evolution of protein families, poses a seeming paradox in light of (a) its chemical similarity, yet (b) dual compatibility with either somatic sex reversal (in the infertile XY daughter) and male development (in the proband’s fertile XY father). A previous study reported no detectable specific DNA-binding activity (34). If valid and extrapolatable *in vivo*, this finding would suggest either (i) that the father exhibited WT germ-line mosaicism; (ii) that the SRY defect could be compensated or bypassed depending on genetic background, or (iii) that the variant SRY gains a non-DNA-mediated function to sustain male gene regulation. Although cryptic mosaicism cannot be excluded, one or both of the latter two suppositions seem plausible. Because the original report relied on the gel mobility-shift assay (GMSA)—susceptible to kinetic artifacts^3^ (40, 80, 81)—our companion study undertook biophysical studies of the WT and Y127F domain-DNA complexes (47). The present FRET-based assays of protein-DNA affinities, an equilibrium technique, have demonstrated that the dissociation constants of WT SRY and Y127F SRY are indistinguishable within experimental error (**Figure 6** and **Table 1**, column 4). Similarly, only a slight difference was observed in DNA bending by PGE or FRET (**Figures 6C**, **7B**; column 6 in **Table 1**). Nonetheless, the mutation attenuates by ca. twofold both occupancy of embryonic testis-specific enhancer elements in the *Sox9/SOX9* gene (**Figure 5** and **Table 1**, column 3) and gene-regulatory activity (columns 2 and 3 in **Table 1**). Because these cellular perturbations are broadly in accordance with previous studies of diverse inherited Swyer alleles (**Supplemental Figure S5** and **Table S3** (30, 31), cryptic germ-line mosaicism need not be invoked to rationalize the phenotype of the proband’s father.

**Figure 8:**
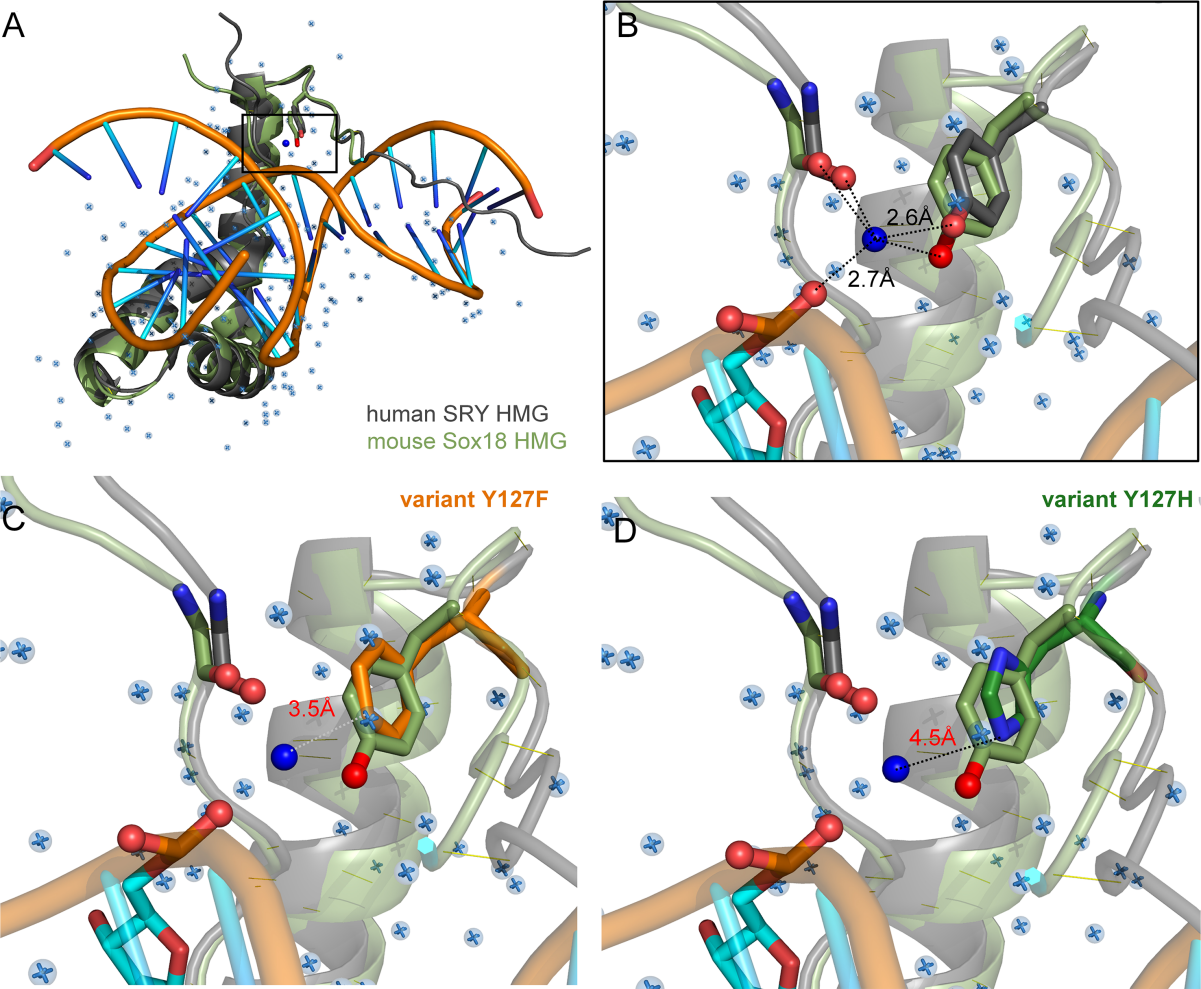
Intervening Y72-anchored water molecule in homologous SOX-DNA interface. **(A)** Structural alignment of human SRY complex (7) and mouse Sox18 HMG box-DNA complex (PDB entries 1J46 and 4Y60, respectively). The Y72-associated water molecule in the Sox18 structure is shown as a dark blue sphere, and other crystallographic water molecules as pale blue spheres. Consensus residue side chains of Y72 are shown as sticks. **(B)** Expanded view of Y72-associated water molecule. Homologous Y21 side chains (SRY in gray, and Sox18 in light green, each with the *para*-hydroxyl in red) occupy similar positions in homologous structures. Measured distance of the para-hydroxyl group to the water molecule is 2.6 Å is each protein and 2.7Å from the water to the DNA phosphate backbone. Distances form the N-terminal carboxyl-group are 3.1 Å and 2.3 Å for Sox18 and human SRY, respectively. For clarity, also shown as sticks are neighboring hydrogen-bond acceptors: a main-chain carbonyl oxygen in the N-terminal β-strand (box residue 5) and a DNA phosphate (C**p**A in bottom DNA strand 5’-(A/C)ACAA(A/T)-3’; underlined). Positions of water molecules were not determined in the NMR-derived structure of the SRY-DNA complex (7). **(C, D)** Rigid-body models of variant protein-DNA interface. **(C)** A water-mediated hydrogen bond would be absent in a Y127F complex (white dotted line) due to removal of the *para*-hydroxyl group. **(D)** Y127H is predicted to exhibit too long a distance from imidazole nitrogen atoms and the DNA to enable side chain-DNA hydrogen bonding (red). .

Tyr and Phe differ only by the presence or absence of a *para*-hydroxyl group in corresponding aromatic rings. It is remarkable that such a subtle chemical modification in a transcription factor could underlie so dramatic a phenotypic outcome as DSD. We imagine that the *para*-hydroxyl group of Y127 functions in some way, not apparent in the present biochemical studies, to enhance the gene-regulatory activity of SRY. In our companion article in this issue (47) we propose that Y127 serves as an *anchor point* for a bound water molecule bridging the protein and DNA surfaces. Although bound water molecules were not sought or identified in the solution structure of the SRY box-DNA complex (as a technical limitation of NMR) (7), a subset of homologous SOX-DNA co-crystal structures exhibits exactly such a bridging water molecule (**Figure 8**). We envision that this bound water molecule, presumably part of a solvation network at this interface, “locks” the bent protein-DNA complex to prolong its kinetic lifetime and enhance the precision of DNA bending. As foreshadowed by studies of the prokaryotic Trp repressor more than 30 years ago (84–86), bound water molecules can be critical elements in protein-DNA recognition. Although the specific DNA-bending properties of WT SRY and Y127F SRY are similar, the more clearly attenuated DNA bending by the Y127C and Y127H domains suggests that this solvated interface contributes to the precision of SRY-directed DNA bending. Such precision is probed in our companion study (47) through time-resolved FRET and distance-distribution analysis of bent DNA complexes (29, 43, 60).

Inherited Swyer Syndrome, although rare, is of conceptual interest as an experiment of nature probing the mechanistic boundaries of SRY function. The subtlety of such mutations (shared by a fertile XY father and sterile XY daughter) honors in the breach Waddington’s Principle of developmental canalization (see **Box 1**) (30, 31, 87, 88). That loss of a single Tyr-anchored water molecule from a protein-DNA interface, as posited here, might render a developmental program ambiguous and would be a striking demonstration of the “hesitant beginnings” of male sex determination (97) as uncovered in intersexual mice with strain-specific B6-Y^POS^ incompatibility (98–100). Inherited Swyer syndrome extends such mouse models to a human genetic switch at the edge of ambiguity. It would be of future interest to extend our ensemble analysis of mean input-output relationships in the SRY-*SOX9* regulatory axis to single-cell analyses of stochastic gene expression (101).

Box 1 **Sex is different: Waddington’s principle in the breach.**

**Figure 9:**
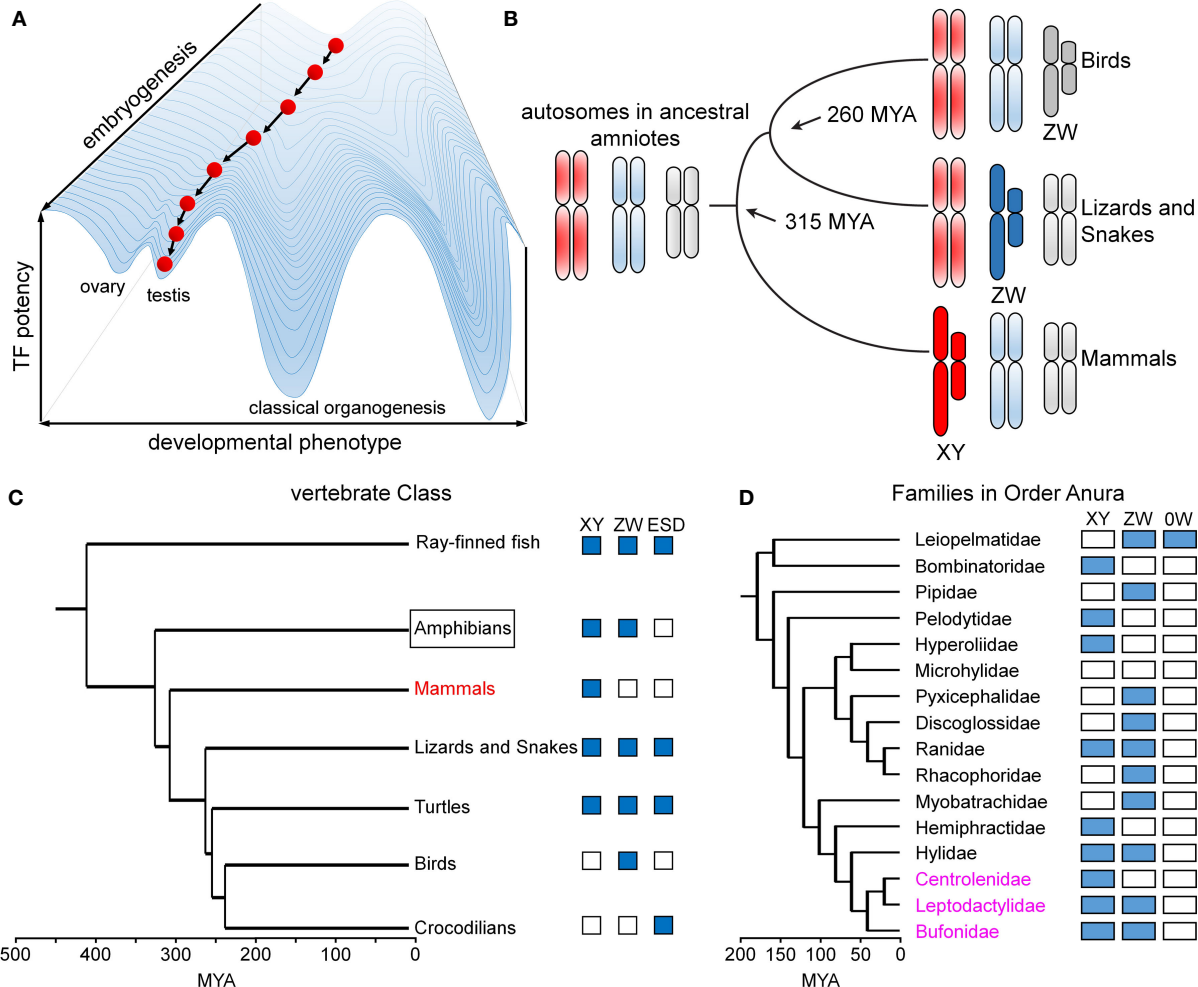
Developmental canalization and fluidity of vertebrate sex determination. **(A)** Waddington’s principle envisions an epigenetic developmental landscape (49). Canalization ensures robust cell-fate determination in organogenesis. This panel was adapted from (50) as differentiating cells (red ball) follow canalized path to predetermined outcome. **(B)** Divergence evolution and re-invention of sex chromosomes as exemplified by multiple independent origins of genetic sex-determining mechanisms in birds (ZW), lizards and snakes (ZW) and mammals (XY). This panel was adapted from (51). **(C)** Phylogeny of vertebrate sex determination (VSD) mechanisms. Boxes indicate distinct sex-determining mechanisms in a given clade; ESD indicates environmental sex determination (52). **(D)** Time-calibrated phylogenetic tree of frog/toad superfamilies (53). Prominent within this clade (Anura) is fluidity of sex-determining systems (XY, ZW, 0W). Highlighted in magenta are three families radiating in South America exhibiting rapid divergence, likely to have been accelerated by changing climates (54). This panel was adapted from (53). Time scales in panels **(C, D)** represent millions of years ago (MYA).

Metazoan development and associated tissue-specific GRNs are ordinarily robust to genetic co-variation and environmental fluctuations. This principle, known as Waddington canalization (87, 88), is enforced by both conserved biochemical mechanisms (49) and the topology of regulatory networks (89). A celebrated example of developmental stability in developmental gene expression is provided by the Hox gene family (90, 91), invariant even among species with varying body plans (91). Similarly, specific transcription factors exhibit analogous functions in rudimentary photoreceptors, compound eyes and vertebrate eyes (92). Such developmental processes and pathways are “canalized” in an epigenetic landscape (**Figure 9A**).

Sex is different. Sex chromosomes come and go rapidly on an evolutionary time scale (**Figure 9B**) (93) in association with striking fluidity among sex-determining systems (**Figure 9C**) (93). We imagine that developmental routes leading to gonads (red ball at bottom left in **Figure 9A**) are less canalized than classical organogenetic programs (bottom right). Attenuated canalization enables innovation: VSD pathways may be regulated by genes (genetic sex determination; GSD) or environment (ESD), even within a single Order, Family, or even Genus. The distinct GSD systems differ in heterogametic sex (XY, WZ, and 0W) (94), with rapid emergence of evolutionary novelty especially prominent among frogs and toads (Order Anura; **Figure 9D**) (53). Intersexual phenotypes are abundant in the wild, especially in the presence of environmental hormone disruptors (54).

Inherited Swyer alleles of SRY provide a model for human male sex determination at the edge of ambiguity. Because such alleles must be compatible with a male phenotype (in the fertile father) and female body plan (in the XY daughter), the extent of functional perturbations to the program of Sertoli-cell differentiation in principle defines the degree of robustness of this program. That this and previous studies of inherited mutations in SRY have uncovered only subtle molecular defects (30, 31) highlights an anomalous failure of canalization, honoring Waddington’s Principle in the breach. In our companion study in this issue, we ascribe the Y127F “father-daughter paradox” to the loss of a single interfacial water molecule. Therein lies the thin thread of mammalian sex determination (95, 96).

## Data Availability Statement

The original contributions presented in the study are included in the article/**Supplementary Material**. Further inquiries can be directed to the corresponding authors.

## Author Contributions

Y-SC co-designed, performed, and interpreted the cell-based studies of SRY function. JR designed, performed, and interpreted the biochemical assays with purified proteins. MW conceived the project, integrated the results, provided structural interpretations, and oversaw preparation of the manuscript. MW is the guarantor of this work and, as such, had full access to all the data in the study and takes responsibility for the integrity of the data and the accuracy of the data analysis. All authors contributed to the article and approved the submitted version.

## Funding

This work was supported in part by the INCITE Scholars Program of the Lilly Foundation and the Distinguished Professors Fund of Indiana University (MW).

## Conflict of Interest

The authors declare that the research was conducted in the absence of any commercial or financial relationships that could be construed as a potential conflict of interest.

## Publisher’s Note

All claims expressed in this article are solely those of the authors and do not necessarily represent those of their affiliated organizations, or those of the publisher, the editors and the reviewers. Any product that may be evaluated in this article, or claim that may be made by its manufacturer, is not guaranteed or endorsed by the publisher.

## Footnotes

1 Genes are designated in italics, and proteins without italics. Primate genes and proteins are capitalized in full (*e.g*., genes *SRY* and *SOX9*; proteins SRY and SOX9), and other mammalian proteins and genes only initial capitalization (*e.g*., genes *Sry* and *Sox9*; proteins Sry and Sox9).

2 Homologous clinical mutations in *SRY* and *SOX* genes give rise to different clinical syndromes depending on the respective biological function of the specific family members. Whereas mutations in SRY, for example, yield DSD phenotypes, mutations in the HMG box of SOX10 are associated with Waardenburg syndrome type II and type IV (also designated Waardenburg-Shah syndrome), characterized by impaired hearing with changes in skin, hair, and eye coloring (78). Mutations in the HMG box of SOX9 cause campomelic dysplasia, characterized by abnormalities of cartilage and bone in variable association with XY DSD (79). Developmental abnormalities of bone are also associated with mutations in SOX4 and SOX11 (77). The diverse SOX-associated genetic syndromes are collectively designated “SOXopathies” (8).

3 Kinetic instability of the variant protein-DNA complex, if present, could have attenuated the shifted band as a known limitation of GMSA (40, 80, 81). In brief, because GMSA is not an equilibrium method, an accelerated rate of dissociation can lead to a diffuse or undetectable shifted band. In such cases, GMSA data can underestimate the degree of residual DNA affinity.
