# Supplementary material for: Tenuous Transcriptional Threshold of Human Sex Determination. I. SRY and Swyer Syndrome at the Edge of Ambiguity
Source: Front Endocrinol (Lausanne). 2022 Jul 26;13:945030. doi: 10.3389/fendo.2022.945030 (PMC9360328; doi:10.3389/fendo.2022.945030)
Supplement: Supplementary file 1 [file DataSheet_1.pdf]

Supplemental Information

for

Tenuous Transcriptional Threshold of Human Sex Determination. I. SRY and Swyer Syndrome at the Edge of Ambiguity

Yen-Shan Chen, Joseph D. Racca, & Michael A. Weiss

Purpose of Supplement

This Supplement, containing five figures and three tables, describes the cell models used in this study and their validation. Our experimental strategy to optimize the level of SRY protein expression in these cells is described, intended to avoid artifacts of protein over-expression otherwise associated with transient-transfection protocols. The molecular logic underlying qPCR-based monitoring of an SRY-directed gene-regulatory network (GRN) is discussed and justified.

Table of Contents

Table of Contents 1
Purpose of Supplement 1
Supplement discussion 2
Figure S1 4
Figure S2 5
Figure S3 6
Figure S4 7
Figure S5 8
Table S1 9
Table S2 11
Table S3 12
References 13

Validation of model cell lines. Selected SRY-responsive cell lines provide a model of the bipotential gonadal ridge. The protein SRY (designated Sry in rodents and other non-primate therian mammals) functions as a stage- and tissue-specific transcription factor in the XY gonadal ridge initiates testis formation via stage- and lineage-specific activation of target gene *SOX9* (designated *Sox9* in rodents and other non-primates) (1). Because such activation is seldom observed among mammalian cell lines in general (**Table S1**), we exploited two exceptional cell lines in which the chromosomal architecture and epigenetic structure of *Sox9* (rat) or *SOX9* (human) enabled such SRY-directed gene regulation (**Figure S1**; (2)): one line (CH34) derived from the bipotential genital ridge of an XY rat embryo (3), the other (LNCaP) fortuitously from a human prostate-cancer derived cell line (4).

In the two SRY-responsive cell lines, the protein binds, as expected, within core elements of two testis-specific enhancer elements (TESCO and Enh13, respectively (1, 5)), 783 and 850 base pairs (bp) upstream of the *SOX9* transcriptional start site. Expression of the SOX9 protein as an architectural transcription factor in turn regulates a ramifying male-specific GRN that recapitulates previous *in vivo* transcriptional profiling of the differentiating XY gonadal ridge in murine embryos (6). Control studies were performed in cell lines *refractory to such activation* (**Figure S1** and **S2**), including an embryonic stage-matched rat cell line also derived from the XY gonadal ridge but lacking pre-Sertoli markers (CH15 cells) (2, 3). We thank Prof. P.K. Donahoe (Massachusetts General Hospital and Harvard Medical School) for cell lines CH34 and CH15.

SRY-directed transcriptional activation of the endogenous *SOX9* gene in LNCaP (human) and of *Sox9* in CH34 (rat) cells was associated in each case with SRY occupancy of enhancer elements TESCO and Enh13 (**Figure S3A**), but not with occupancy of far-upstream element XYSR (7) (**Figure S3B**) or of other potential SRY-binding sites in this chromosomal region. Activation required specific DNA binding as demonstrated by the inactivity of SRY variants containing substitutions that impair such binding (I68A and I68T; the cantilever position of the HMG box (8-10)), which serve as controls in our transcriptional responses assays.

Cell-based transcription assays. The genetic function of SRY in the embryonic gonadal ridge requires occupancy of *Sox9/SOX9* enhancer sites, critical to the assembly of Enh13- and TESCO complexes as stage- and tissue-specific enhanceosomes (5, 11, 12). Use of the orthologous rat and human genes in their respective native chromosomal locations as complementary functional read-outs of WT or variant SRYs circumvents long-established artifacts of reporter-gene assays (such as a reporter plasmid-encoded *luciferase* gene in a standard co-transfection assay (13)), often exacerbated by effects of marked transcription factor overexpression ($>10^6$ molecules/cell, as typically occurs in transient-transfection assays due to use of strong viral promoters) (14, 15).

Our experimental approach required construction or screening of special cell lines in which the epigenetic structure of *Sox9/SOX9* enables SRY-directed transcriptional activation. This feature of embryonic pre-Sertoli cells is uncommon among standard mammalian cell lines, and so transient transfection of SRY does not ordinarily lead to enhanced transcription of *Sox9/SOX9*. In the case of embryonic rat pre-Sertoli cell line CH34, SRY-responsiveness was sought and obtained by design: microdissection of the bipotential XY gonadal ridge just prior to its morphologic differentiation by Dr. Patricia Donahoe (MGH and Harvard Medical School) (9). In the case of human LNCaP cells, such responsiveness was obtained by serendipity, following the systematic screening of >12 established XY mammalian lines. Fortuitously, in both rat CH34

cells and in human LNCaP cells, the orthologous *Sox9/SOX9* genes were found to exhibit similar patterns of epigenetic histone marks. Cell lines CH34 and LNCaP thus each permitted assessment of SRY gene-regulatory activity through quantitative reverse-transcriptase (rt)-PCR (qPCR) assays, focusing on relative mRNA abundances of *Sox9/SOX9* and other downstream genes in the testis-specific GRN as defined *in vivo* transcriptional profiling (16).

A key aspect of our protocol is expression of the transfected epitope-tagged SRY *in the physiological range* (*i.e.*, 10^3-10^4 molecules/cell (17))—a marked reduction relative to conventional protocols leading (**Table S2**). SRY expression level can be "tuned" downward by two approaches:

(i) *Classical method*. Dilution of expression plasmid by an excess parent plasmid (typically 50-fold dilution) maintains total plasmid mass, but modulates the fraction of the transfected plasmids able to express the protein of interest (15, 18).

(ii) *Ligand-regulated transcriptional activation*. An exogenous ligand may be exploited to regulate an engineered operon via a specific ligand-binding gene-regulatory protein. A popular such system uses the antibiotics tetracycline (Tet) or doxycycline (DOX) as ligands permeable to mammalian cells but not ordinarily found in vertebrates.

To validate the robustness of these complementary methods, we extended our plasmid-dilution studies (2, 15) using a Tet-*on* system (illustrated in schematic form in **Figure S4-A**); this system in principle provides a more precise approach to expression tuning than plasmid dilution (19). If the expressed protein, like SRY in pre-Sertoli cells, functions as transcriptional "control knob" to activate a downstream gene-regulatory network, then ligand-based adjustments in expressed protein concentration (in mean molecules per cell) would provide quantitative insight into network activation (**Figure S4-B**).

In the present study *Sox9* expression were monitored at a similar range of intracellular SRY concentrations, whether tuned by the Tet-on system or by plasmid dilution (see main-text **Figure 6**). Tet-*on*-regulated CH34 cells respectively treated with successive DOX concentrations (0, 100, 200, 400 and 800 ng/mL) induced SRY expression to a similar extent as plasmid-dilution ratios of 50X, 25X, 5X, and 1X (no dilution).

The "proof is in the pudding" summarized in **Figure 6C-E** (main text). Whereas mean SRY expression levels >10^5 molecules per cell (mean mol/cell in the population) led, as an artifact, to progressive increases in *Sox9* mRNA accumulation (blue-shaded region at right **Figure 6C, E**), a transcriptional <u>plateau</u> was observed between 10^3-10^5 SRY mol/cell. In functional assessment of how mutations in SRY affect its intrinsic gene-regulatory activity, "apples and apples" requires similar expression levels in this plateau range. The importance of this principle was highlighted in studies of I90M SRY when expressed in the physiological range (15) versus under conditions of marked over-expression (13).

Residue Numbering. Clinical mutations in human SRY are ordinarily given in relation to residue numbers in the full-length protein. For clarity, consensus positions in the HMG box are also given; e.g., Y127 in full-length SRY is residue 72 in an HMG box consensus (20).

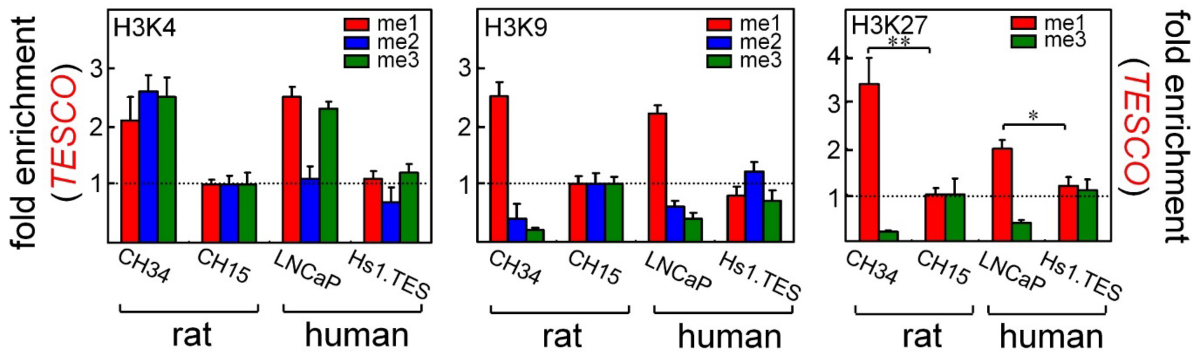

Figure S1 | Epigenetic histone marks within the N-terminal arm of histone 3. (left) Mono-, di-, and tri-methylation of Lys4 (modifications me1, me2 or me3 in H3K4; respectively shown in red, blue and green). Fold-enrichment in TESCO (ChIP primer set a) was evaluated in non-transfected cell lines CH34, CH15, LNCaP and Hs1.TES; (middle) corresponding ChIP analysis of mono-, di-, and tri-methylation of Lys9; (right) ChIP analysis of mono- and tri-methylation of Lys27. CH34- and LNCaP cells exhibited activating marks at H3K4, H3K9 and H3K27 with reduction in repressive mark me3 at H3K9; the other two cell lines (non-responsive to transfected SRY) by contrast exhibited attenuated activating marks and repressive marks at H3K27.

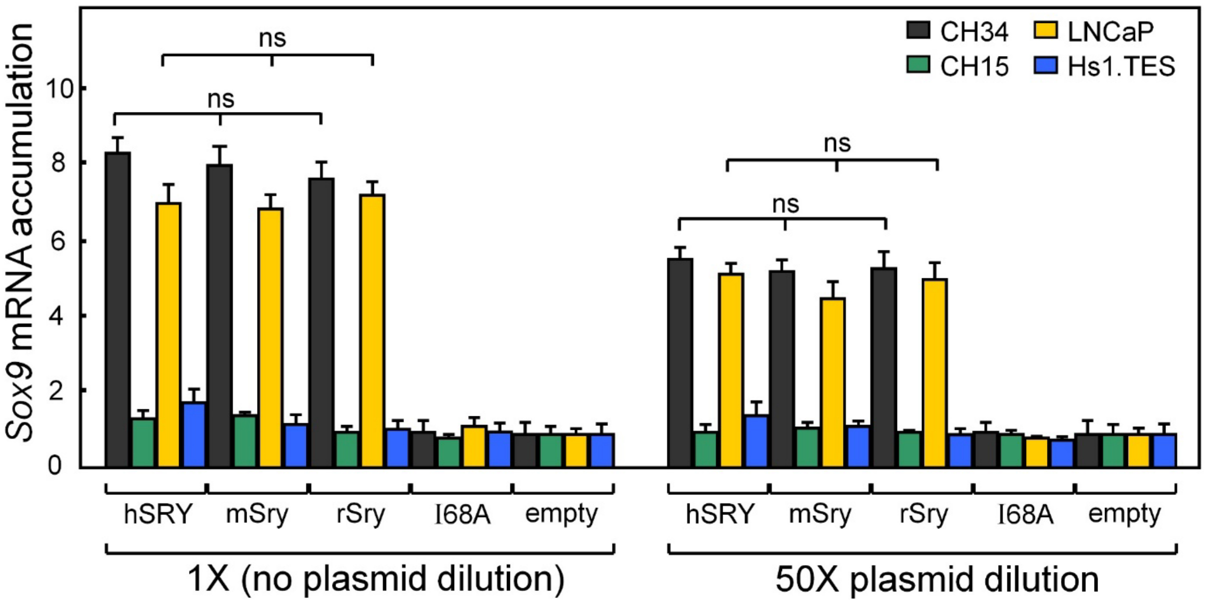

Figure S2│ SRY-dependent transcriptional activation of the endogenous *Sox9/SOX9* gene. The expression of genes was probed following transient transfection of SRY-expression constructs in two rat- and two human cell lines: (black and green) rat embryonic CH34 pre-Sertoli cells and control fibroblast-like CH15 cells (green bars) (3); (gold and blue) human prostate-cancer-derived cell line LNCaP and fetal Leydig cell line Hs1.TES. Transfected constructs encoded human SRY (hSRY), mouse Sry (mSry), rat Sry (rSry) or an inactive mutant human SRY (I68A (10)). Sox9/SOX9 mRNA accumulation was probed by qPCR under both 1X and 50X plasmid-dilution transfection conditions (left- and right-hand histograms), a protocol designed to modulate the mean number of expressed SRY molecules per cell. SRY-directed transcriptional activation of *SOX9/Sox9* was observed in CH34 and LNCaP cells, but not in CH15 or Hs1.TES cells.

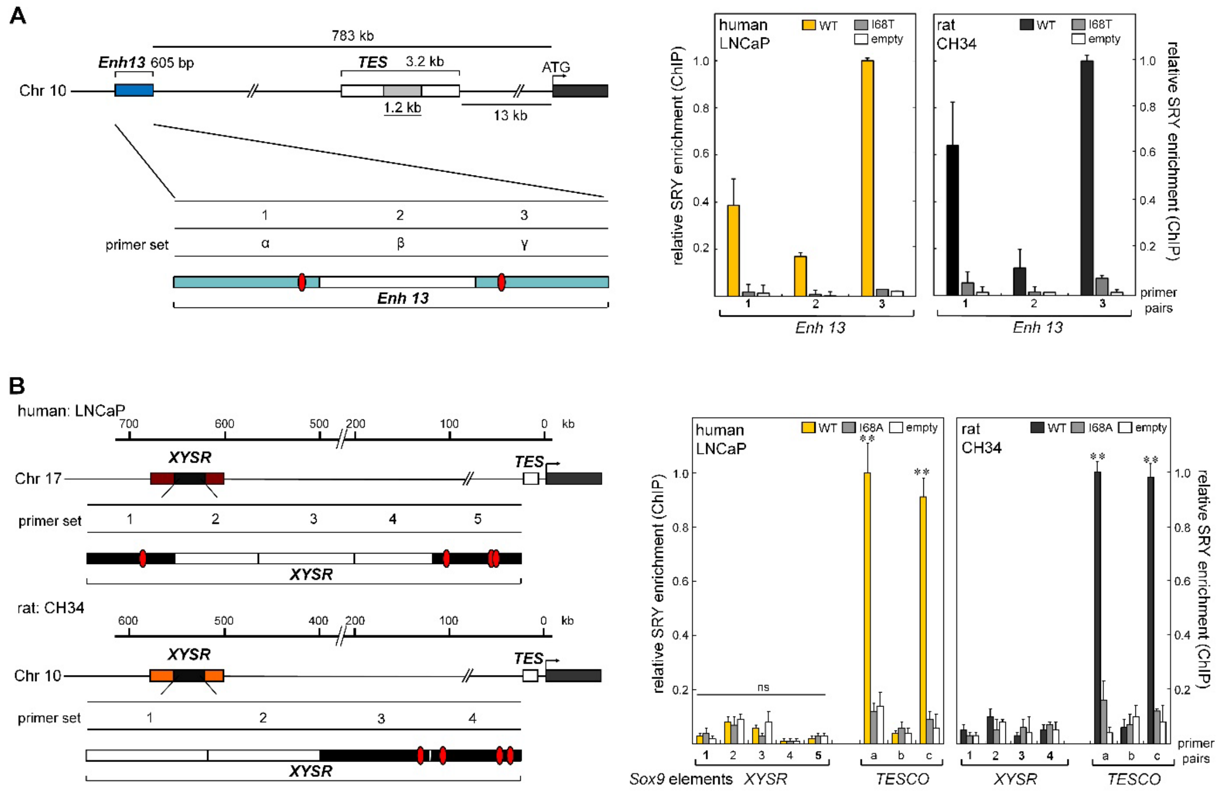

Figure S3|Cellular models of the bipotential gonadal ridge at the edge of morphologic differentiation. (**A**) Left: rat *Sox9* gene with a far upstream enhancer element (*Enh13*) (5); *TES* and *TES* core elements (*TESCO*) are in light gray. Potential SRY binding sites are predicted in *Enh13* fragments 1 and 3 (primer sets α and γ). Right: In lines CH34 and LNCaP transient transfection of SRY led to its occupancy of sites within *Enh13* as quantified in histograms: (yellow and black bars), WT SRY; (gray and white bars) negative controls I68A SRY (8, 9)) and empty plasmid. SRY binding sites are predicted in *Enh13* fragments 1 and 3. (**B**) Left: Testis-specific enhancer element (*TES*) in human *SOX9* and rat *Sox9* gene (1); *XYSR* element is also shown (7). Right: Histograms describing occupancy of SRY binding sites within *TESCO* or *XYSR* (7): (yellow and black bars) WT SRY; (gray and white bars) negative controls as in panel A. Despite absence of observed occupancies in *XYSR*, potential SRY binding sites are predicted in human upstream *SOX9* fragments 1 and 5 (bold beneath histogram) and in rat *Sox9* upstream fragments 3 and 4 (bold).

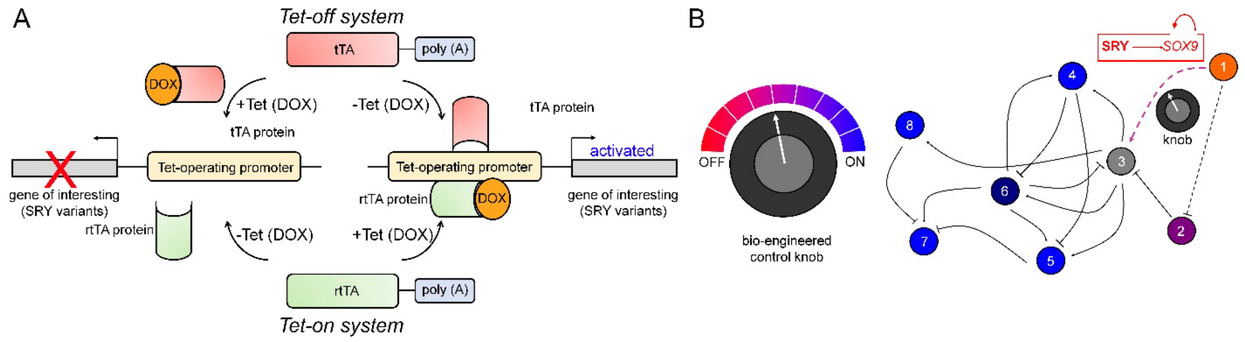

Figure S4| The *Tet-on* system enables ligand-dependent expression of SRY. (**A**) Schematic mechanism of complementary Tet-on/off systems. Upper panel, **Tet-off** system exploits tetracycline (Tet) or doxycycline (DOX) as a ligand to prevent the tTA transcription factor from binding to specific DNA sites at the promoter. Transcriptional expression of the protein-coding region interesting is thereby inhibited. Lower panel, **Tet-on** system likewise exploits Tet or DOX to bind the rtTA transcription factor, but in this case enabling the DOX-bound rtTA protein to bind the promoter. Gene expression (herein, WT or variant SRY coding regions) is activated. By appropriately setting the concentration of DOX, extent of protein expression (herein, SRY) can be "tuned" to achieve the desired mean number of protein molecules per cell. In the present study, the Tet-on system was employed to corroborate the plasmid-dilution protocol. (**B**) Schematic "tuning" of a gene-regulatory network by an engineered "control knob" (inset at upper right). The set point of a biochemical switch may be designed to modulate the strength of critical connections in schematic network (dashed lines). The tuned circuit could be SRY-*Sox9* activation (red box) in mammalian sex determination: from node 1 (orange) to node 2 (purple; inhibitory) and to node 3 (gray; stimulating).

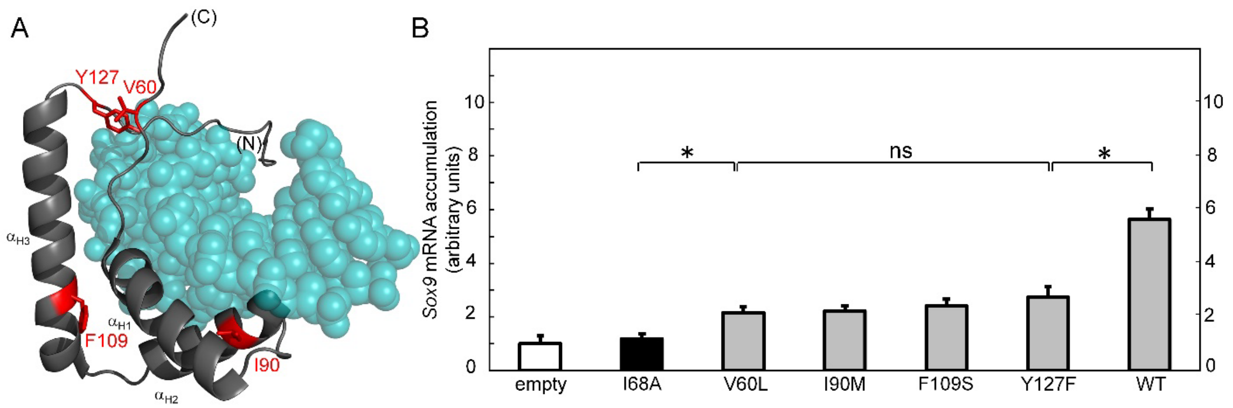

Figure S5│ Diverse inherited mutations in the HMG box of human SRY define a general twofold transcriptional threshold for robust male sex determination. (**A**) NMR-derived structure of the L-shape HMG box of human SRY (gray ribbon) and DNA (space-filling model; adjure). Three inherited clinical mutations with known function activities are highlighted as red side chains. Residue numbers refer to the full-length human SRY sequence; subtract 55 to obtain consensus box positions. (**B**) Histogram presents the transcriptional activities of the inherited mutation and WT SRY, which were probed by their *Sox9* activation in rat pre-Sertoli CH34 cells. Substitution I68A (black; box "cantilever" position 13), devoid of specific DNA-binding activity (10), provided a negative control (15). Inherited mutations exhibit similar 2-fold reduction comparing to the activity of WT SRY. Statistical comparisons: (*) Wilcox p-values <0.05 whereas the "ns" indicates no significant difference (p-values > 0.05).

Table S1. Master Table: Cell Lines and Properties

species	cell lines	source (gender)	transcription activation marks at TES[a]	transcription repression marks at TES[a]	SOX9/Sox9 response[b]	ChIP TESCO	ChIP XYSR[c]
human	**LNCaP**	prostate cancer (male)	+	-	7-fold	+	-
	Hs 1.Tes	normal testis (male)	-	+	ND[d]	-	-
	HEK 293T	embryonic kidney (female)	-	+	ND	-	-
	NT2-D1	testis derived from lung cancer (male)	-	+	ND	-	
monkey	COS-7	monkey kidney tissue (male)	-	+	ND	N/A	N/A
mouse	NIH 3T3	embryo fibroblast (male)	-	+	ND	-	
	MEF	embryo fibroblast (mixed)			ND		
rat	**CH34**	embryonic rat gonadal ridge (male)	+	-	8-fold	+	-

CH15	embryonic rat gonadal ridge (male)	-	+	ND	-	-

[a]Defined by the histone epigenetic profile: methylation status on H3K4, H3K9, and H3K27 at TES element.
[b]Tested by regular transient transfection protocol: 1μg of SRY-encoded plasmid with CMV promoter per 10^6 cells.
[c]Far-upstream element XYSR is potentially associated with XY female sex reversal phenotype (7)
[d]ND, not detected due to apparent abolishment of endogenous SOX9/Sox9 activation after transfection

Table S2. Effect of plasmid dilution on HA-tagged SRY expression in CH34 cells[a]

SRY-encoded plasmid dilution	wild type SRY			F109S SRY			F109S w/MG132		
	1X	10X	50X	1X	10X	50X	1X	10X	50X
relative SRY signal (on gel)	6.7	2.6	1.7	2.1	1	-	6.1	2.8	1.9
loading protein dilution	100X	100X	1X	1X	1X	1X	100X	100X	1X
relative SRY signal (normalized)	670	260	1.7	2.1	1	0.15	610	280	1.9
molecular copy numbers of SRY	7×10^5	3×10^5	~1900	~2200	~1100	~170	6×10^5	3×10^5	~2000

[a]The summary table describes expression of transfected SRY signal strength relative to tubulin as a function of plasmid dilution; CH34 cells were transfected without dilution ("1X"). Protein expression is reduced by 390-fold following 50-fold (50X) plasmid dilution. The estimation was taken from a single gel and blot. Because of the greater abundance of the 1X transfected WT SRY-encoded plasmid, some of the total cellular protein extracts were diluted by 100-fold ("100X") for loading into gel to obtain bands of similar intensity ("-" indicates the signal is too weak to be analyzed on a blot image).

Technical note. Plasmid dilution in CH34 cells enables an appropriate intracellular concentration of SRY to be achieved as follows. The method to compare the numbers of SRY and its variant copies in CH34 cell was described previously. Briefly, a typical mammalian cell contains 300 pg of total protein, and has a tubulin content about 3% of total protein. Western blot intensities enabled estimation of the relative SRY accumulation in cells by a four-step calculation.

(This procedure assumes that the affinities of the anti-HA and anti-tubulin antisera are similar as indicated by the vendor; our qualitative conclusions are robust to fivefold errors.)

Step 1. We assumed that a typical mammalian cell contains by weight the following amount of tubulin: 300×10^{-12} grams x 3%= 9×10^{-12} grams;

Step 2. We next estimated the number of tubulin molecules (molecular weight 110 kDa) as [9×10^{-12} grams/110×10^3 grams per mole] x 6.02×10^{23} molecules per mole = 5×10^7 molecules;

Step 3. We in turn estimated the number of transfected SRY molecules per cell in the 1X transfection using its Western-blot signal strength relative to α-tubulin: 5×10^7/100 (the tubulin-blotting dilution ratio)/2.5 (relative band intensity: tubulin:1X SRY signal)/28.9% (transfection efficiency), which yields an estimate of 7×10^5 molecules (which are predominantly in the nucleus);

Step 4. Finally, we estimated the number of transfected SRY molecules per cell following 50-fold plasmid dilution using relative signal strength between 1X and 50X HA-SRY Western blots: 7×10^5 molecules/390, which yields an estimate of near 1900 molecules per cell. This degree of expression is within the middle of the range of cellular abundances expected of a lineage- and stage-specific transcription factor (10^2-10^4 molecules per nucleus).

Table S3. Inherited Swyer mutations in human SRY

Mutations	CGD/PGD	Ovotestis	clinical features<br>Gonadal blastoma	Mosaic father	Sister-brother	location	*Sox9* activation	Mechanism	ref
V60L	CGD			■		minor wing	46±7 (%)	impaired nuclear localization	(15)
I90M	CGD			■	■	major wing α2	52±6 (%)	impaired nuclear export	(15)
F109S	CGD			■	■	major wing α3	51±8 (%)	accelerated protein degradation	(2)
Y127F	CGD			■		minor wing	61 ± 12 (%)	water-mediated DNA-interaction	(21)

References

1. Sekido R, Lovell-Badge R. Sex Determination Involves Synergistic Action of Sry and Sf1 on a Specific *Sox9* Enhancer. *Nature* (2008) 453:930-4.
2. Racca JD, Chen Y-S, Yang Y, Phillips NB, Weiss MA. Human Sex Determination at the Edge of Ambiguity Inherited Xy Sex Reversal Due to Enhanced Ubiquitination and Proteasomal Degradation of a Master Transcription Factor. *J Biol Chem* (2016) 291(42):22173-95.
3. Haqq CM, Donahoe PK. Regulation of Sexual Dimorphism in Mammals. *Physiol Rev* (1998) 78:1-33.
4. Horoszewicz JS, Leong SS, Kawinski E, Karr JP, Rosenthal H, Chu TM, et al. LNCaP Model of Human Prostatic Carcinoma. *Cancer Res* (1983) 43(4):1809-18.
5. Gonen N, Futtner CR, Wood S, Garcia-Moreno SA, Salamone IM, Samson SC, et al. Sex Reversal Following Deletion of a Single Distal Enhancer of Sox9. *Science* (2018) 360(6396):1469-73.
6. Wilhelm D, Hiramatsu R, Mizusaki H, Widjaja L, Combes AN, Kanai Y, et al. Sox9 Regulates Prostaglandin D Synthase Gene Transcription *in Vivo* to Ensure Testis Development. *J Biol Chem* (2007) 282(14):10553-60.
7. Kim GJ, Sock E, Buchberger A, Just W, Denzer F, Hoepffner W, et al. Copy Number Variation of Two Separate Regulatory Regions Upstream of Sox9 Causes Isolated 46,Xy or 46,Xx Disorder of Sex Development. *J Med Genet* (2015). Epub 2015/01/22. doi: jmedgenet-2014-102864 [pii]10.1136/jmedgenet-2014-102864.
8. King CY, Weiss MA. The Sry High-Mobility-Group Box Recognizes DNA by Partial Intercalation in the Minor Groove: A Topological Mechanism of Sequence Specificity. *Proc Natl Acad Sci USA* (1993) 90:11990-4.
9. Haqq CM, King CY, Ukiyama E, Falsafi S, Haqq TN, Donahoe PK, et al. Molecular Basis of Mammalian Sexual Determination: Activation of Müllerian Inhibiting Substance Gene Expression by Sry. *Science* (1994) 266:1494-500.
10. Weiss MA, Ukiyama E, King CY. The Sry Cantilever Motif Discriminates between Sequence- and Structure- Specific DNA Recognition: Alanine Mutagenesis of an Hmg Box. *J Biomol Struct Dyn* (1997) 15:177-84.
11. Croft B, Ohnesorg T, Hewitt J, Bowles J, Quinn A, Tan J, et al. Human Sex Reversal Is Caused by Duplication or Deletion of Core Enhancers Upstream of Sox9. *Nat Commun* (2018) 9(1):5319.
12. Atlas G, Sreenivasan R, Sinclair A. Targeting the Non-Coding Genome for the Diagnosis of Disorders of Sex Development. *Sex Dev* (2021):1-19.
13. Pontiggia A, Rimini R, Harley VR, Goodfellow PN, Lovell-Badge R, Bianchi ME. Sex-Reversing Mutations Affect the Architecture of Sry-DNA Complexes. *EMBO J* (1994) 13:6115-24.
14. Berk AJ, Boyer TG, Kapanidis AN, Ebright RH, Kobayashi NN, Horn PJ, et al. Mechanisms of Viral Activators. *Cold Spring Harb Symp Quant Biol* (1998) 63:243-52. Epub 1999/06/29.
15. Chen YS, Racca JD, Phillips NB, Weiss MA. Inherited Human Sex Reversal Due to Impaired Nucleocytoplasmic Trafficking of Sry Defines a Male Transcriptional Threshold. *Proc Natl Acad Sci USA* (2013) 110(38):E3567-76. Epub 2013 Sep 3. doi: 10.1073/pnas.1300828110.
16. Xu Z, Gao X, He Y, Ju J, Zhang M, Liu R, et al. Synergistic Effect of Sry and Its Direct Target, Wdr5, on Sox9 Expression. *PLoS One* (2012) 7(4):e34327. Epub 2012/04/24. doi: 10.1371/journal.pone.0034327.
17. Hiller G, Weber K. Radioimmunoassay for Tubulin: A Quantitative Comparison of the Tubulin Content of Different Established Tissue Culture Cells and Tissues. *Cell* (1978) 14(4):795-804. Epub 1978/08/01.
18. Chen YS, Racca JD, Sequiera PW, Phillips NB, Weiss MA. Microsatellite-Encoded Domain in Rodent Sry Functions as a Genetic Capacitor to Enable the Rapid Evolution of Biological Novelty *Proc Natl Acad Sci USA* (2013) 110(33):E3061-70. Epub 2013 Jul 30. doi: 10.1073/pnas.1300860110.

19. T Das A, Tenenbaum L, Berkhout B. Tet-on Systems for Doxycycline-Inducible Gene Expression. *Curr Gene Ther* (2016) 16(3):156-67.
20. Murphy EC, Zhurkin VB, Louis JM, Cornilescu G, Clore GM. Structural Basis for Sry-Dependent 46-X,Y Sex Reversal: Modulation of DNA Bending by a Naturally Occuring Point Mutation. *J Mol Biol* (2001) 312(3):481-99.
21. Racca JD, Chatterjee D, Chen Y-S, Yang Y, Georgiadis M, Haas E, et al. Tenuous Transcriptional Threshold of Human Sex Determination. Ii. Sry Exploits a Water-Mediated DNA Clamp. *Front Endocrinol* (2022).
